# Rotational Spectroscopy and Partial Semi-Experimental Equilibrium (*r_e_* ^SE^) and Substitution (*r_s_* ^SE^) Structures of Cyclobutenone (*c*‑C_4_H_4_O)

**DOI:** 10.1021/acs.jpca.6c04937

**Published:** 2026-09-28

**Authors:** Brian J. Esselman, Michael J. Carrillo, Madeleine G. Atwood, Nitai P. Sahoo, Zoe K. Deems, Michael J. Tubergen, Maria A. Zdanovskaia, R. Claude Woods, Robert J. McMahon

**Affiliations:** † Department of Chemistry, 5228 University of Wisconsin−Madison, Madison, Wisconsin 53706, United States; ‡ Department of Chemistry and Biochemistry, 4229 Kent State University, Kent, Ohio 44240, United States; § Quantum Theory Project, Departments of Physics and Chemistry, University of Florida, Gainesville, Florida 32611, United States

## Abstract

Partial semi-experimental equilibrium (*r*_*e*_^SE^) and substitution (*r*_*s*_^SE^) structures of cyclobutenone (*c*-C_4_H_4_O, *C*_*s*_, *μ*_*a*_ = 3.4 D) have been determined for the first time via rotational spectroscopy. The rotational spectrum of this reactive species, which contains a highly strained four-membered ring, has been measured from 9 to 735 GHz. Over 3800 transitions for the ground vibrational state of the normal isotopologue of cyclobutenone were measured, assigned, and least-squares fit to sextic distorted-rotor A- and S-reduced Hamiltonians in the I^*r*^ representation, providing a complete set of quartic and sextic centrifugal distortion parameters. Rotational transitions for each of the singly substituted heavy-atom isotopologues were measured at natural abundance. Rotational constants (*B*_0_) for the six isotopologues were converted to equilibrium values (*B*_*e*_) using CCSD­(T)/cc-pCVTZ vibration-rotation interaction and electron-mass corrections, enabling the determination of partial *r*_*e*_^SE^ and *r*_*s*_^SE^ structures that define all of the heavy-atom parameters of cyclobutenone. The two different sets of semi-experimental structural parameters (*r*_*e*_^SE^ and *r*_*s*_^SE^) are compared to the “best theoretical estimate” (BTE *r*_*e*_) values, which are derived from the CCSD­(T)/cc-pCV5Z *r*_*e*_ structure with additional corrections addressing finite basis set, higher-level electron correlation, and relativistic effects, as well as incorporating the diagonal Born–Oppenheimer correction. Additionally, transitions of the four lowest-energy vibrationally excited states have been assigned, measured, and least-squares fit to A-reduced, sextic centrifugally distorted-rotor Hamiltonians in the I^*r*^ representation. While ν_21_ was well-modeled using a single-state Hamiltonian, 2ν_21_, ν_14_, and ν_20_ form an anharmonic- and Coriolis-coupled triad and were treated by a three-state Hamiltonian model.

## Graphical abstract

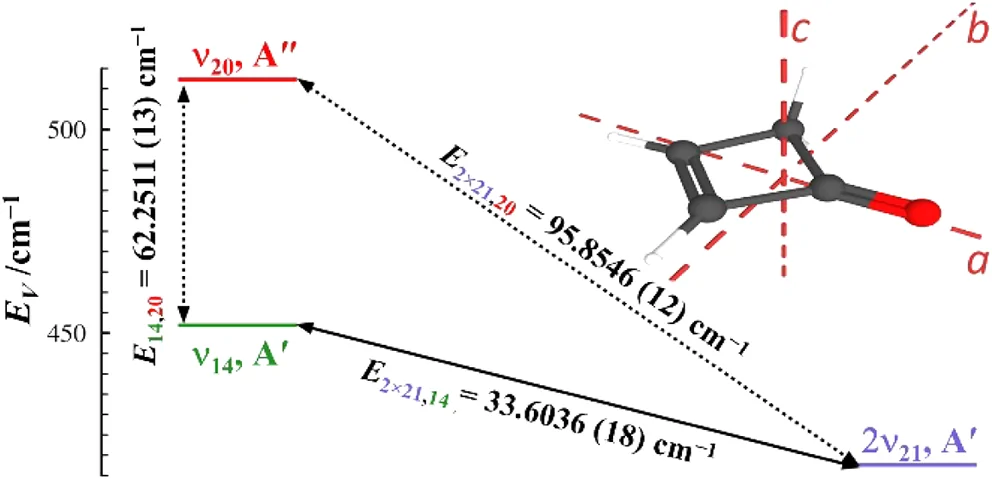

## Introduction

Cyclobutenone, an unsaturated, four-membered ring ketone (*c*-C_4_H_4_O, *C*_*s*_, κ = −0.659, *μ*_*a*_ = 3.4 D, *μ*_*b*_ = 0.1 D;^1^ Figure 1), is a higher-energy isomer of furan and vinylketene (energies relative to furan = 0 kcal/mol; vinylketene = 12 kcal/mol; cyclobutenone = 18 kcal/mol)^2^ with strain energy of approximately 28 kcal/mol.^3–6^ Largely due to the ring strain of having three *sp* ^2^-hybridized carbon atoms in a four-membered ring, synthesizing and obtaining a pure sample of cyclobutenone for spectroscopic study has presented a substantial challenge.^7–11^ When isolated in pure form, cyclobutenone polymerizes within 24 h, even when held at −78 °C,^9^ but it is stable in chloroform solution at 0 °C for prolonged periods.^10,11^ Danishefsky and coworkers synthesized cyclobutenone in a two-step process from 3-oxocyclobutane-1-carboxylic acid,^10,11^ a procedure that enabled Carrillo *et al*. to obtain its microwave spectrum for the first time.^1^ In these experiments, cyclobutenone was used promptly after synthesis and purification due to its propensity to rapidly polymerize and the desire to avoid chloroform or other volatile solvents that would complicate the gas-phase spectroscopy. Analysis of the microwave spectrum established a planar ring geometry,^1^ which was expected from prior theoretical investigations and the presence of the conjugated π_C–O_ and π_C–C_ bonds within the small ring.^2,12,13^ The planar ring structure with two out-of-plane hydrogen atoms is consistent with the absence of observable tunneling splitting of the microwave signals that would arise from an equilibrium of two equivalent, non-planar (puckered) ring structures. This assignment was further supported by comparing the out-of-plane second moment (*P*_*cc* 0_ = 1.608 uÅ^2^) of cyclobutenone to theoretical values and to those of other ring-containing molecules.

**Figure 1:**
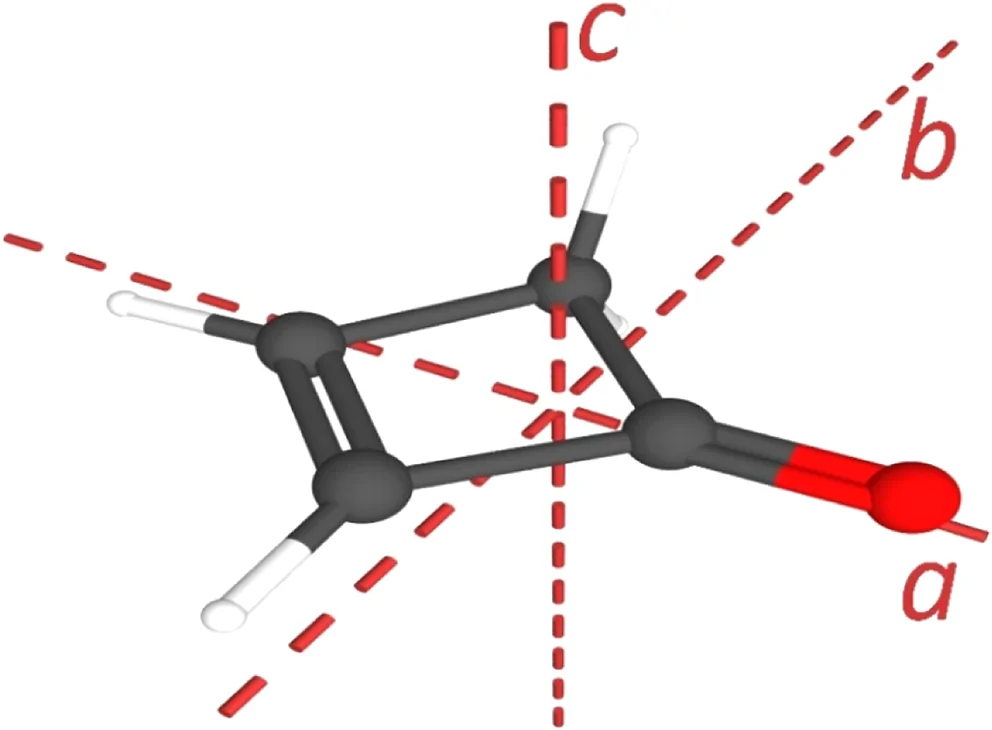
Cyclobutenone structure (*c*-C_4_H_4_O, *C_s_, κ* = −0.659, *μ_a_* = 3.4 D, *μ*_*b*_ = 0.1 D)^1^ with principal inertial axes.

In this work, we present the rotational spectroscopy of cyclobutenone and its heavy-atom isotopologues from 9 to 735 GHz. A modified procedure for sample preparation and storage greatly enhances the lifetime of cyclobutenone, enabling data collection for a prolonged period (several weeks) and even shipping of the sample between institutions. The improved sample preparation and subsequent new spectroscopy provided sufficient information to determine partial semi-experimental equilibrium (*r*_*e*_^SE^) and partial semi-experimental substitution (*r*_*s*_^SE^) structures of cyclobutenone. The structural parameters are compared to the “best theoretical estimate” (BTE *r*_*e*_) values, which are derived from the CCSD­(T)/cc-pCV5Z optimized geometry (*r*_*e*_) with additional corrections addressing finite basis set, higher-level electron correlation, and relativistic effects, as well as incorporating the diagonal Born–Oppenheimer correction. Although cyclobutenone is a higher-energy isomer on the C_4_H_4_O potential energy surface, it is a conformationally rigid, polar molecule with a structure that is related to cyclopropenone, a known interstellar molecule.^14^ The spectroscopic data reported herein, which cover the majority of the spectral range of modern radiotelescopes, provide the basis for astronomical searches for this molecule.

## Methods

### Synthetic Methods

A commercial sample of 3-bromocyclobutanone was treated with the low-volatility base tri-*n*-butylamine, which enabled the isolation of neat cyclobutenone by distillation (Scheme 1).^9–11^ Cyclobutenone was subsequently dissolved in diethyl carbitol,^9^ which enabled us to store it for several weeks and ship it between institutions. Diethyl carbitol proved to be an ideal solvent for use with gas-phase experiments because of its low volatility. Under vacuum, a solution of cyclobutenone in diethyl carbitol at room temperature affords a convenient vapor pressure of cyclobutenone without interfering solvent signals (*vide infra*). Details concerning the preparation and characterization of cyclobutenone are provided in Supporting Information.

**Scheme 1:**
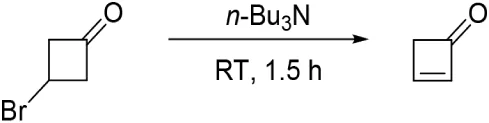
Cyclobutenone Synthesis

a Based upon previous work of Sieja^9^ and Danishefsky and coworkers.^10,11^

### Experimental Methods

The rotational spectrum of cyclobutenone was collected using two different spectrometers: (1) a broadband millimeter-wave rotational spectrometer at the University of Wisconsin–Madison with a frequency range of 82 to 750 GHz and (2) a high-resolution, jet-cooled molecular beam cavity-based Fourier-transform microwave (FTMW) spectrometer with a frequency range of 9 to 22 GHz at Kent State University. Both the millimeter-wave and microwave spectrometers have been described previously.^15,16^ At UW–Madison, the nearly complete spectrum from 85 to 735 GHz was obtained in an automated fashion over approximately 14 days, given the following experimental parameters: 0.6 MHz/s sweep rate, 10 ms time constant, and 50 kHz AM and 500 kHz FM modulation in a tone-burst design.^17^ The vapor pressure of cyclobutenone above diethyl carbitol was sufficient to collect these data at pressures ranging from 5 to 15 mTorr at room temperature. Using diethyl carbitol as the storage solvent, the rate of polymerization was slowed sufficiently that, even after the 14-day data collection, cyclobutenone continued to be released by the solution and the solution had obtained only a pale-yellow coloration (from initially being colorless). Millimeter-wave data acquired in this work assumes a uniform 50 kHz frequency measurement uncertainty. To obtain the microwave spectrum at Kent State, the cyclobutenone sample in diethyl carbitol was placed in a reservoir nozzle and carried into the Fabry–Pérot resonator with Ar gas at a 1.5 atm backing pressure, which produced a supersonic jet expansion in the vacuum chamber. Each gas pulse is 800 μs and is followed by a 0.2 μs microwave irradiation pulse. The heterodyne detection circuit is closed after an additional 2 μs delay, and the signal is digitized at 100 MHz for 400 μs. A nominal measurement uncertainty of 3 kHz is assumed for all microwave transitions. The individual spectroscopic segments were stitched together and analyzed using Kisiel’s Assignment and Analysis of Broadband Spectra (AABS) software.^18,19^ Spectroscopic data were analyzed using SPFIT and SPCAT,^20^ as well as PIFORM, AC, PLANM, and other useful helper programs.^21^ All output files from the least-squares fits are provided in Supporting Information.

### Computational Methods

The computational methods employed in this work are similar to those used previously for a number of structure determinations.^15,22–31^ A development version of CFOUR^32^ was used for geometry optimizations at the ae-CCSD­(T) level of theory using cc-pCVTZ, cc-pCVQZ, and cc-pCV5Z basis sets,^33^ as well as using fc-CCSDT­(Q)/cc-pVDZ, fc-CCSD­(T)/cc-pVDZ, and SCF/PVTZ.^34,35^ Additional optimizations using SCF/cc-pVTZ with the DBOC=ON keyword and CCSD­(T)/cc-pCVTZ with the RELATIVISTIC=X2C1E keyword were carried out. Together, these optimizations comprise the calculations necessary for the “best theoretical estimate” (BTE), which uses the CCSD­(T)/cc-pCV5Z structure as a basis with four corrections that are evaluated using conventional methodology:^36–38^

Residual basis set effects beyond cc-pCV5Z.
Residual electron correlation effects beyond the CCSD­(T) treatment.
Effects of scalar (mass-velocity and Darwin) relativistic effects.
The diagonal Born-Oppenheimer correction (DBOC), calculated for the normal isotopic species, *c*-C_4_H_4_O.

The equations used to calculate these corrections, the computational methodology employed, the values of the corrections, and all output files are available in Supporting Information.

A second-order vibrational perturbation theory (VPT2) anharmonic frequency calculation evaluating the cubic force constants using analytical second derivatives at displaced points,^39–41^ based on the CCSD­(T)/cc-pCVTZ optimized structure, provided vibration-rotation interaction constants (*B*_0_ – *B*_*e*_) and quartic and sextic centrifugal distortion constants for each isotopologue. Rotational *g*-tensor calculations at the same level of theory provided electron-mass corrections for each isotopologue. Computed, isotopologue-specific centrifugal distortion constants were used in the least-squares fits where constants could not be determined experimentally.

A partial *r*_*e*_^SE^ structure was determined in an analogous manner to that used in previous studies.^15,22–31^ The computed vibration-rotation interaction corrections, the electron-mass corrections, and the averaged determinable constants $\left(B_{0}^{\prime \prime }\right)$ ^42^ from the A- and S-reduction fits were input into the *xrefit* module of CFOUR. The *xrefit* module generates equilibrium rotational constants, which, after conversion to the corresponding moments of inertia, are used to determine the *r*_*e*_^SE^ structural parameters via a nonlinear least-squares fit. A partial *r*_*s*_^SE^ structure was determined using Kraitchman’s analysis.^43,44^

## Results and Discussion

### Rotational Spectra of the Normal Isotopologue

Cyclobutenone (*c*-C_4_H_4_O, *C*_*s*_) is a moderately prolate asymmetric top (*μ*_*a*_ = 3.30 D, *μ*_*b*_ = 0.05 D, *κ* = −0.660; CCSD­(T)/cc-pCVQZ, this work) with a spectrum dominated by ^*a*^R_0,1_ transitions, which have a room-temperature intensity peak at approximately 380 GHz (Figure 2). Observation of the cyclobutenone millimeter-wave spectrum enables assignment, measurement, and least-squares fitting of transitions at much higher frequencies and with much higher *J* and *K*_*a*_ quantum number values than observed in previous work.^1^ In the millimeter-frequency range, the low-*K*_*a*_ series of the ^*a*^R-branches are readily assigned by Loomis-Wood plots from either computed spectroscopic constants or experimental rotational constants from the previous microwave study,^1^ supplemented with computed centrifugal distortion constants. The most prominent features in Figure 3 are low-*K*_*a*_^*a*^R_0,1_ transitions, which form bands that progress to lower frequency with increasing *K*_*a*_, turn around after several transitions, and progress to higher frequency with increasing *K*_*a*_. Initially, these transitions are degenerate pairs of ^*a*^R_0,1_ transitions with different *K*_*a*_ values and the same *K*_*c*_ value. As *K*_*a*_ increases, they lose degeneracy and at high *K*_*a*_ values become degenerate with transitions sharing the *K*_*a*_ value. These degenerate pairs of high-*K*_*a*_^*a*^R_0,1_ transitions are responsible for the smaller bands of transitions in Figure 3 that increase smoothly in intensity from low to high frequency. Most of the unassigned transitions in Figure 3 are due to vibrationally excited states higher in energy than those described herein. Analysis of those higher-energy vibrationally excited states is ongoing, but beyond the scope of the current article.

**Figure 2:**
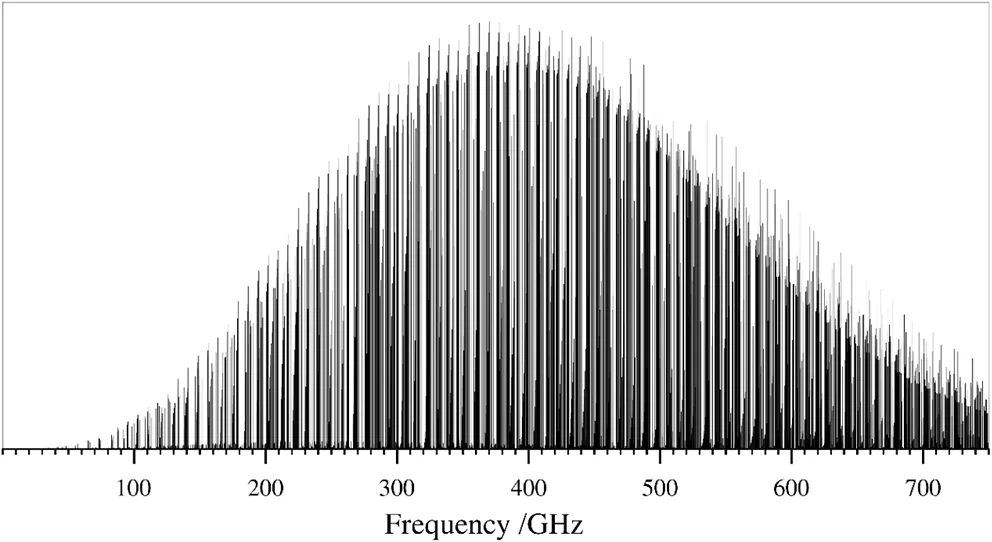
Predicted rotational spectrum of the ground vibrational state of the normal isotopologue of cyclobutenone at 300 K from the spectroscopic constants determined in this work.

**Figure 3:**
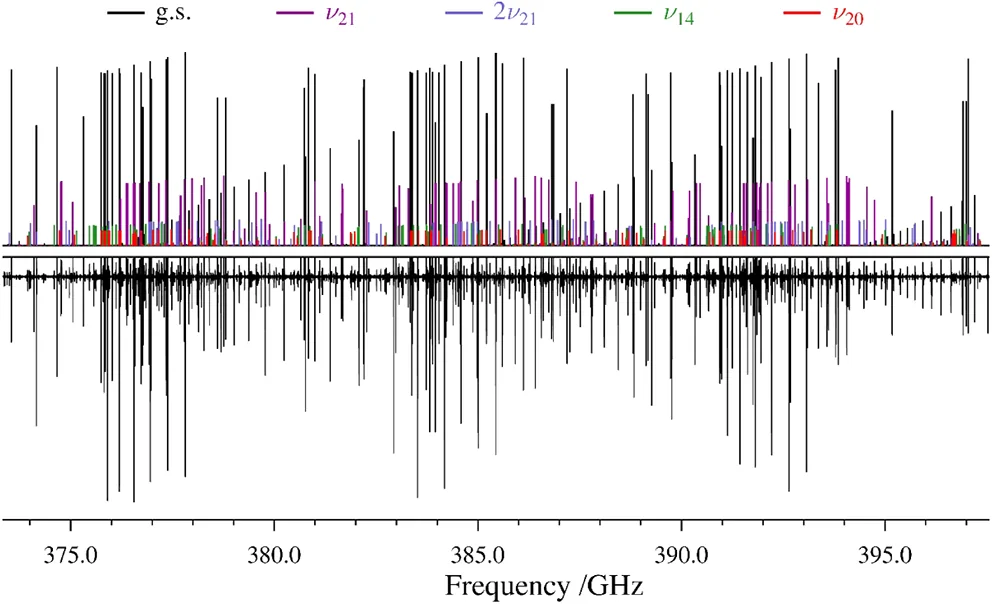
Experimental rotational spectrum (bottom) of cyclobutenone from 373.5 to 397.5 GHz and stick spectra (top) of the ground (black), ν_21_ (purple), 2ν_21_ (slate blue), ν_14_ (green), and ν_20_ (red) vibrational states from experimental spectroscopic constants. Unassigned transitions are attributed to higher-energy vibrationally excited states.

As shown in the data distribution plot (Figure 4), the final data set includes transitions of *J*″ up to 95 and *K*_*a*_″ up to 54. The favorable combination of spectral density, frequency coverage, and transition intensities enabled ^*a*^R_0,1_ transitions of cyclobutenone to be measured for nearly every value of *K*_*a*_″ from *J*″ 20 to 50. Due to the lower intensity of the ^*a*^Q-branch transitions and the spectral density, far fewer of these transitions were able to be measured and included in the data set. Since the range of *J*″ and *K*_*a*_″ values of the energy levels involved in the R-branch transitions encompasses the range of *J*″ and *K*_*a*_″ values of the energy levels involved in the Q-branch transitions, the Q-branch transitions make minimal contribution to the values of the parameters determined by the least-squares fit. Two of the Q-branch transitions from the microwave region of the spectrum have *obs*. – *calc*. values greater than 3× their nominal measurement uncertainty (3σ) when least-squares fit with the millimeter-wave data, reflecting larger uncertainties than previously assigned, arising from poorly resolved line shapes for these transitions.

**Figure 4:**
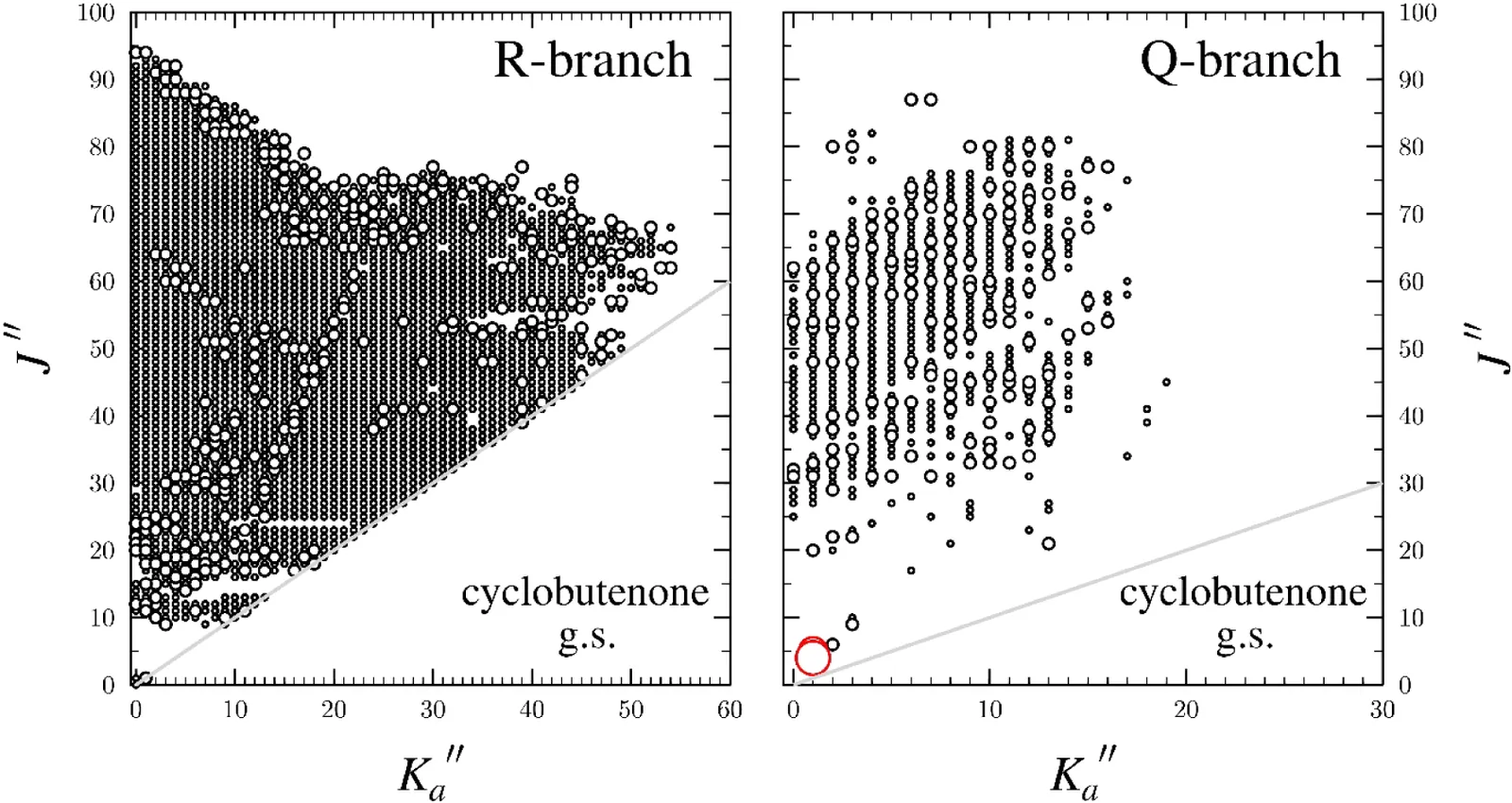
Data distribution plot for the least-squares fit of spectroscopic data for the vibrational ground state, normal isotopologue of cyclobutenone. The size of the outlined circle is proportional to the value of |(*f*_obs._ – *f*_calc._)/δ*f*|, where δ*f* is the frequency measurement uncertainty. Two of the quotient values are equal to or greater than 3× their nominal measurement uncertainty (3σ) and are shown in red. The data set includes all of the microwave transitions from Carrillo *et al*.,^1^ two of which are the red transitions.

The rotational transitions represented in Figure 4 have been least-squares fit to A- and S-reduced sextic centrifugally distorted Hamiltonians in the I^*r*^ representation (σ_fit_ = 31 kHz). The values of the resulting spectroscopic constants, those from the previous microwave work,^1^ and their corresponding computed values (CCSD­(T)/cc-pCVTZ) are provided in Table 1. The current data set provides sufficient information to determine a complete set of spectroscopic constants in both the A and S reductions, though with a large relative uncertainty in the S-reduction constant *H*_*K*_, 0.0033(12) Hz. Holding *H*_*K*_ at its computed value in the least-squares fit did not improve the agreement between the other spectroscopic constants and their computed values or improve the σ_fit_ value. As expected, the agreement between the previous rotational constants determined only from the microwave work and those from this study is excellent. The agreement of the centrifugal distortion constants, of course, is less satisfactory, due to the much smaller impact of centrifugal distortion at low frequency. The CCSD­(T)/cc-pCVTZ spectroscopic constants show the expected excellent agreement, within 0.6% difference from experiment in the rotational constants and within 5% for the quartic centrifugal distortion constants. The deviation of theory from experiment is much greater for the sextic centrifugal distortion constants, with differences up to 62% for *H*_*JK*_. For reasons that are not entirely obvious, with *κ* = −0.659 170 and nearly identical σ_fit_ values, the quartic centrifugal distortion constants show better agreement between theory and experiment in the S reduction, while the sextic centrifugal distortion constants show better agreement between theory and experiment in the A reduction.

**Table 1:** Experimental and Computed Spectroscopic Constants for the Vibrational Ground State, Normal Isotopologue of Cyclobutenone

	S reduction, I^*r*^ representation				A reduction, I^*r*^ representation		
	Previous Microwave^1^	Current Work^a^			Previous Microwave^1^	Current^a^	
	Experimental	Experimental	CCSD(T)^b^		Experimental	Experimental	CCSD(T)^b^
*A*_0_ (MHz)	12548.248 (14)	12548.269 63 (16)	12483	*A*_0_ (MHz)	12548.256 (14)	12548.268 56 (16)	12483
*B*_0_ (MHz)	5307.560 (3)	5307.559 753 (31)	5276	*B*_0_ (MHz)	5307.567 (3)	5307.567 214 (32)	5276
*C*_0_ (MHz)	3820.178 (3)	3820.176 505 (28)	3798	*C*_0_ (MHz)	3820.171 (3)	3820.169 613 (29)	3798
*D*_*J*_ (kHz)	0.7 (3)	0.579 237 1 (80)	0.563 0	*Δ*_*J*_ (kHz)	0.9 (4)	0.7464 252 (89)	0.723 2
*D*_*JK*_ (kHz)	6.4 (9)	6.477 489 (37)	6.319	*Δ*_*JK*_ (kHz)	5.4 (4)	5.474 062 (37)	5.358
*D*_*K*_ (kHz)	[1.30]^c^	1.489 57 (82)	1.520	*Δ*_*K*_ (kHz)	[2.13]^c^	2.325 24 (84)	2.321
*d*_1_ (kHz)	–0.197 (9)	–0.199 404 8 (32)	–0.193 8	*δ*_*J*_ (kHz)	0.197 (9)	0.199 407 4 (32)	0.193 8
*d*_2_ (kHz)	[−0.0834]^c^	–0.083 601 3 (12)	–0.080 10	*δ*_*K*_ (kHz)	[3.62]^c^	3.590 471 (51)	3.473
*H*_*J*_ (Hz)		0.000 056 94 (75)	0.000 024 10	*Φ*_*J*_ (Hz)		0.000 182 1 (10)	0.000 152 2
*H*_*JK*_ (Hz)		–0.001 170 (13)	–0.000 446 8	*Φ*_*JK*_ (Hz)		0.005 181 (16)	0.005 514
*H*_*KJ*_ (Hz)		0.009 740 (40)	0.007 711	*Φ*_*KJ*_ (Hz)		–0.013 509 (57)	–0.014 08
*H*_*K*_ (Hz)		0.003 3 (12)	0.002 316	*Φ*_*K*_ (Hz)		0.019 5 (12)	0.018 02
*h*_1_ (Hz)		0.000 047 49 (44)	0.000 040 50	*ϕ*_*J*_ (Hz)		0.000 089 70 (46)	0.000 079 30
*h*_2_ (Hz)		0.000 062 87 (28)	0.000 064 10	*ϕ*_*JK*_ (Hz)		0.003 082 (12)	0.003 168
*h*_3_ (Hz)		0.000 042 010 (83)	0.000 038 80	*ϕ*_*K*_ (Hz)		0.036 40(10)	0.034 29
*N*_lines_ ^d^	9	3848		*N*_lines_ ^d^	9	3848	
σ_fit_ (MHz)	0.0027	0.031		σ_fit_ (MHz)	0.002 7	0.031	
$P_{cc_{0}}$ (uÅ^2^)^e^	1.600785(63)	1.600 726 (1)	1.605	$P_{cc_{0}}$ (uÅ^2^)^e^	1.600 588 (63)	1.600 542 (1)	1.605
*κ*	–0.659173	–0.659 173	–0.659 6	*κ*	–0.659 170	–0.659 170	–0.659 6

a Includes transitions from ref 1.

b Evaluated using the cc-pCVTZ basis set.

c Computed at the B3LYP/aug-cc-pVTZ level of theory in ref 1.

d Number of independent transitions.

e *P*_*cc* 0_ *=* −(*I*_*c*_ – *I*_*a*_ – *I*_*b*_)/2 = −(1/2)*Δ*_*i* 0_, calculated using PLANM.

### Rotational Spectra of the Heavy-Atom Isotopologues of Cyclobutenone

The transitions of all singly substituted ^13^C- and ^18^O-isotopologues of cyclobutenone are observable at their natural isotopic abundance. In a similar manner to the normal isotopologue, the rotational transitions were least-squares fit to A- and S-reduced Hamiltonians in the I^*r*^ representation, with centrifugal distortion up to the sextic level (Tables 2 and S2–S7). All of the sextic centrifugal distortion constants were held at their computed values, due to the lower transition counts available for these isotopologues. In addition, quartic terms *Δ*_*K*_, *D*_*K*_, *δ*_*K*_, and *d*_2_ could not be determined for [^18^O]-cyclobutenone and were held at their computed values in the least-squares fitting. The initial fitting of these isotopologues was completed in the millimeter-wave region. With excellent predictions of their microwave transitions from these initial fits, we were able to measure the microwave transitions of the heavy-atom isotopologues for the first time. This was particularly important for [^18^O]-cyclobutenone, where the transitions in the millimeter-wave region were unable to deconvolute *A*_0_ and *B*_0_. Due to the very low intensity of this isotopologue at natural abundance and the spectral density, which is exacerbated by vibrationally excited states at room temperature, no millimeter-wave transitions could be assigned beyond the degenerate low-*K*_*a*_^*a*^R_0,1_ transitions with identical *K*_*c*_. As a result, the three non-degenerate microwave transitions (Figure S6) proved necessary to obtain precise and accurate *A*_0_ and *B*_0_ constants for the [^18^O]-isotopologue for subsequent use in the *r*_*e*_^SE^ and *r*_*s*_^SE^ structure determinations (*vide infra*).

**Table 2:** Experimental Spectroscopic Constants for Cyclobutenone Isotopologues (S Reduction, I*^r^* Representation) *^a^*

	[1-^13^C]-	[2-^13^C]-	[3-^13^C]-	[4-^13^C]-	[1-^18^O]-
*A*_0_ (MHz)	12548.769 4 (26)	12219.063 6 (20)	12546.367 1 (24)	12187.436 1 (20)	12547.805 (48)
*B*_0_ (MHz)	5290.946 58 (16)	5291.741 97 (15)	5189.187 70 (20)	5292.241 48 (17)	4993.401 7 (14)
*C*_0_ (MHz)	3811.627 91 (11)	3780.988 45 (10)	3758.317 06 (12)	3778.187 00 (12)	3654.596 49 (48)
*D*_*J*_ (kHz)	0.582 110 (27)	0.566 293 (18)	0.560 073 (26)	0.568 478 (18)	0.574 7 (43)
*D*_*JK*_ (kHz)	6.391 95 (15)	6.367 40 (12)	6.133 45 (13)	6.430 32 (12)	5.176 (65)
*D*_*K*_ (kHz)	1.554 2 (95)	1.208 0 (46)	1.871 2 (61)	1.183 3 (42)	[1.980]^a^
*d*_1_ (kHz)	–0.199 344 (15)	–0.200 466 (12)	–0.188 466 (18)	–0.201 221 (12)	–0.192 5 (22)
*d*_2_ (kHz)	–0.082 366 2 (75)	–0.086 170 0 (70)	–0.076 403 0 (78)	–0.087 173 7 (75)	[−0.066 43]^a^
*H*_*J*_ (Hz)	[0.000 027 4]	[0.000 020 3]	[0.000 028 1]	[0.000 020 9]	[0.000 020 5]
*H*_*JK*_ (Hz)	[−0.000 545 1]	[−0.000 416 4]	[−0.000 323 6]	[−0.000 669 7]	[−0.000 275 3]
*H*_*KJ*_ (Hz)	[0.007 462]	[0.007 631]	[0.006 132]	[0.008 410]	[0.006 730]
*H*_*K*_ (Hz)	[0.002 646]	[0.001 846 4]	[0.003 777]	[0.001 162]	[0.003 117]
*h*_1_ (Hz)	[0.000 041 6]	[0.000 038 7]	[0.000 041 2]	[0.000 036 9]	[0.000 034 8]
*h*_2_ (Hz)	[0.000 062 4]	[0.000 065 6]	[0.000 058 9]	[0.000 063 8]	[0.000 052 2]
*h*_3_ (Hz)	[0.000 037 8]	[0.000 040 4]	[0.000 033 8]	[0.000 041 3]	[0.000 03]
*N*_lines_ ^b^	649	646	628	663	57
σ_fit_ (MHz)	0.043	0.043	0.045	0.045	0.045
*P*_*cc* 0_ (uÅ^2^)^c^	1.601 064 (5)	1.600 009 (4)	1.601 090 (5)	1.599 614 (5)	1.599 897 (79)
*κ*	–0.661 372	–0.641 920	–0.674 360	–0.639 907	–0.698 915

a Values in brackets held constant at its CCSD­(T)/cc-pCVTZ value.

b Number of independent transitions.

c *P*_*cc* 0_ *=* −(*I*_*c*_ – *I*_*a*_ – *I*_*b*_)/2 = −(1/2)*Δ*_*i* 0_. Calculated using PLANM.

### Partial Semi-Experimental Equilibrium (*r_e_* ^SE^) and Substitution (*r_s_* ^SE^) Structures of Cyclobutenone

As described above, the measured rotational transitions for each isotopologue of cyclobutenone were least-squares fit in the I^*r*^ representation using both the A and S reductions. In a manner consistent with other recent *r*_*e*_^SE^ structure determinations, the experimentally determined spectroscopic constants (*B*_0_^(A)^ or *B*_0_^(S)^) are converted to their reduction-free, or determinable, constants (*B*_0_″) using Equations S1–S6.^42^ Ideally, the *B*_0_″ constants have identical values regardless of representation. In practice, using a well-measured and analyzed data set, the typical extent of agreement between the *B*_0_″ constants matches the statistical uncertainties of the experimental rotational constants (between 0.050 and 0.000 030 MHz). For example, the |*B*_0_″^(A)^ – *B*_0_″^(S)^| values for the normal isotopologue are 0.000 13 MHz, 0.000 06 MHz, and 0.000 05 MHz for *A*_0_, *B*_0_, and *C*_0_, respectively (Table S8). Even for [^18^O]-cyclobutenone, where only 57 transitions are included in the data set, the |*B*_0_″^(A)^ – *B*_0_″^(S)^| values are quite small: 0.012 MHz, 0.000 33 MHz, and 0.000 14 MHz for *A*_0_, *B*_0_, and *C*_0_, respectively. In both of these cases, we are confident that the data is being treated well by both reductions because the |*B*_0_″^(A)^ – *B*_0_″^(S)^| values are the same order of magnitude as the statistical uncertainty in the rotational parameters from their respective least-squares fits.

In structure determinations of nonplanar molecules, like cyclobutenone or cyclopropanone,^31^ where a pair or pairs of hydrogen atoms lie symmetrically above and below the heavy-atom plane of the ring, the values of the uncorrected and corrected *c*-axis planar moments (*P*_*cc* 0_ and *P*_*cc e*_, respectively) provide some indication of the quality of the rotational constants. The *P*_*cc* 0_ values for cyclobutenone are determined using the *B*_0_″ constants of each isotopologue (Table 3). These values should be very similar for all isotopologues with only ^1^H containing species, but not identical due to isotopic dependence of the vibration-rotation interaction. When the *B*_0_″ constants are corrected for the vibration-rotation interaction and electron-mass distribution to obtain the *B*_*e*_ constants, the resulting *P*_*cc e*_ values should be identical for isotopologues with only heavy-atom substitution. In practice, these values are never precisely identical (*e*.*g*. cyclopropanone^31^) but they should show much closer agreement than their corresponding *P*_*cc* 0_ values. The excellent agreement of the fully corrected *P*_*cc e*_ values for cyclobutenone (Table 3, column 5) validates the quality of rotational constants used in the structure determination.

**Table 3:** *c*-Axis Planar Moments (*P_cc_*) of Cyclobutenone Isotopologues with and without Vibration-Rotation and Electron-Mass Corrections *^a^*

Isotopologue	*N*_lines_	*P*_*cc* 0_ (uÅ^2^)^b^	*P*_*cc e*_ (uÅ^2^)^c^	*P*_*cc e*_ (uÅ^2^)^d^	*c*_*H*_ (Å)^e^
Cyclobutenone	3848	1.600 80	1.607 26	1.604 68	0.892 25
[1-^13^C]-	649	1.601 13	1.607 03	1.604 45	0.892 19
[2-^13^C]-	646	1.600 08	1.606 99	1.604 42	0.892 18
[3-^13^C]-	628	1.601 16	1.606 99	1.604 42	0.892 18
[4-^13^C]-	663	1.599 69	1.606 99	1.604 41	0.892 18
[^18^O]-	57	1.599 97	1.606 87	1.604 29	0.892 14
**Average (x̅)**		**1.600 47**	**1.607 02**	**1.604 44**	**0.892 18**
**Std. Dev. (** ***s*** **)**		**0.000 64**	**0.000 13**	**0.000 13**	**0.000 04**

a *P*_*cc* 0_ *=* −(*I*_*c*_ – *I*_*a*_ – *I*_*b*_)/2 = −(1/2)*Δ*_*i* 0_.

b Determined from the averaged *B*_0_″ values.

c Determined from *B*_e_ values with corrections for vibration-rotation interaction.

d Determined from *B*_e_ values with corrections for vibration-rotation interaction and electron-mass distribution.

e *c*-coordinate of the H atom; *c*_H_ = (*P*_*cc e*_ */*2*m*_H_)^1/2^ = distance from the *a, b*-plane (heavy-atom plane); *m*_H_ = 1.007 825 035 u for ^1^H.

From the *B*_*e*_ values, partial semi-experimental equilibrium (*r*_*e*_^SE^) and substitution (*r*_*s*_^SE^) structures for cyclobutenone have been determined. The structural parameters for the *r*_*e*_^SE^ structure, the *r*_*s*_^SE^ structure, and the *r*_*e*_ BTE values are provided in Table 4 and Figure 5. Without substitution of each of the ^1^H atoms, reliable structural parameters cannot be determined for parameters involving a hydrogen atom. Nevertheless, *r*_*e*_^SE^ and *r*_*s*_^SE^ structural parameters have been determined for the remaining heavy-atom framework. In the case of the *r*_*e*_^SE^ structure, the parameters involving hydrogen atoms have been held constant at their BTE values. Without a complete structure, however, it is not possible to determine if the *r*_*e*_^SE^ or *r*_*e*_ BTE values for any parameter are more reliable. Given the very close agreement shown in previous works between the BTE parameters calculated in a similar fashion and the parameters determined from a complete *r*_*e*_^SE^ structure,^10,17–26^ use of the BTE parameters should minimize the impact of holding these parameters at a constant value during the least-squares fitting of the values of the determined structural parameters. To test this assumption, an *r*_*e*_^SE^ structure was also determined holding the unfit parameters at their CCSD­(T)/cc-pCV5Z values. The resulting structure afforded residuals in *A*_*e*_, *B*_*e*_, and *C*_*e*_ an order of magnitude larger than those from the *r*_*e*_^SE^ structure obtained using the BTE parameter values (Table S9). This outcome provides additional support for the validity of the BTE analysis, which involves the application of four corrections mentioned above to the CCSD­(T)/cc-pCV5Z *r*_*e*_ structure. It is clear, however, that using the BTE parameter values for all parameters involving hydrogen atoms did not afford structural parameters that display the typical agreement expected between *r*_*e*_ BTE and *r*_*e*_^SE^ structural parameters obtained using experimental data sets that include isotopologues that substitute each atomic position, or many more than a single-substitution set of isotopologues. It might be expected that the most reliable parameter in the partial *r*_*e*_^SE^ structure is *R*_O–C1_, because it is the only parameter that involves atoms that are not directly bonded to hydrogen. The BTE value of the *R*_O–C1_ distance, however, does not fall within 2σ of the semi-experimental equilibrium value. In contrast, the *r*_*e*_^SE^ value is in excellent agreement with the *R*_O–C1_ distance determined by the *r*_*s*_^SE^ (*vide infra*). The *r*_*e*_^SE^ values *R*_C1–C2_ and *R*_C2–C3_ are in agreement with their BTE values, though in the case of *R*_C1–C2_ it is at least partially due to the large statistical uncertainty (Table 4). The ring angles are in agreement with their BTE values, with the exception of *θ*_C2–C1–C4_, which has the smallest uncertainty. The angles involving the oxygen atom (*θ*_O–C1–C2_ and *θ*_O–C1–C4_) are in agreement with their BTE values only because of their unusually large statistical uncertainties. These large uncertainties are likely due to the proximity of both C1 and O to the *a*-axis. It is likely that inclusion of isotopologues that rotate the principal axes further would help better determine the C1 and O positions, in addition to removing the need to hold parameters constant in the least-squares fitting. The rotational constants for the [2-^2^H]- and [2,3-^2^H]-isotopologue families are expected to be most useful based upon their expected rotations of the principal axes (inferred from their predicted dipole moments). The C3–C4 distance shows a larger discrepancy from its BTE value than is typical, which may be related to the proximity of the C3 atom to the *a*-axis. Given the difficulty of interpreting a partial semi-experimental structure, no definitive conclusions can be drawn at this time.

**Table 4:** Experimental and Computational Structural Parameters of Cyclobutenone

	*r*_*s*_^SE^ ^a^	*r*_*e*_^SE^ ^a^	CCSD(T) BTE	CCSD(T)/cc-pCV5Z
*R*_O–C1_ (Å)	1.197 5	1.197 6 (1)	1.198 1	1.197 1
*R*_C1–C2_ (Å)	1.492 5	1.491 4 (14)	1.492 4	1.492 6
*R*_C1–C4_ (Å)	1.555 3	1.556 5 (14)	1.556 6	1.556 1
*R*_C2–C3_ (Å)	1.348 8	1.348 3 (6)	1.348 3	1.347 7
*R*_C3–C4_ (Å)	1.520 5	*1.521 0 (5)* ^b^	1.523 1	1.521 5
*R*_C2–H_ (Å)		[1.079 0]	1.079 0	1.078 9
*R*_C3–H_ (Å)		[1.079 1]	1.079 1	1.079 1
*R*_C4–H_ (Å)		[1.089 0]	1.089 0	1.088 8
*θ*_O–C1–C2_ (°)	136.519	136.54 (19)	136.480	136.499
*θ*_O–C1–C4_ (°)	134.008	133.99 (19)	134.011	134.053
*θ*_C2–C1–C4_ (°)	89.473	*89.473 (6)* ^b^	89.509	89.448
*θ*_C1–C2–C3_ (°)	91.196	91.256 (33)	91.260	91.255
*θ*_C1–C4–C3_ (°)	82.713	*82.648 (35)* ^b^	82.620	82.670
*θ*_C2–C3–C4_ (°)	96.618	96.623 (49)^c^	96.610	96.628
*θ*_H–C2–C1_ (°)		[134.333]	134.333	134.398
*θ*_H–C4–C1_ (°)		[114.656]	114.656	114.670
*θ*_H–C3–C2_ (°)		[132.264]	132.264	132.267
*θ*_H–C4–C1–O_ (°)		[64.353]	64.353	64.352

a Vibration-rotation interaction and electron-mass corrections computed at the CCSD­(T)/cc-pCVTZ geometry.

b Value in italics is a dependent parameter determined in an alternate *Z*-matrix.

c Value determined from other parameters using mathematical relationships.

**Figure 5:**
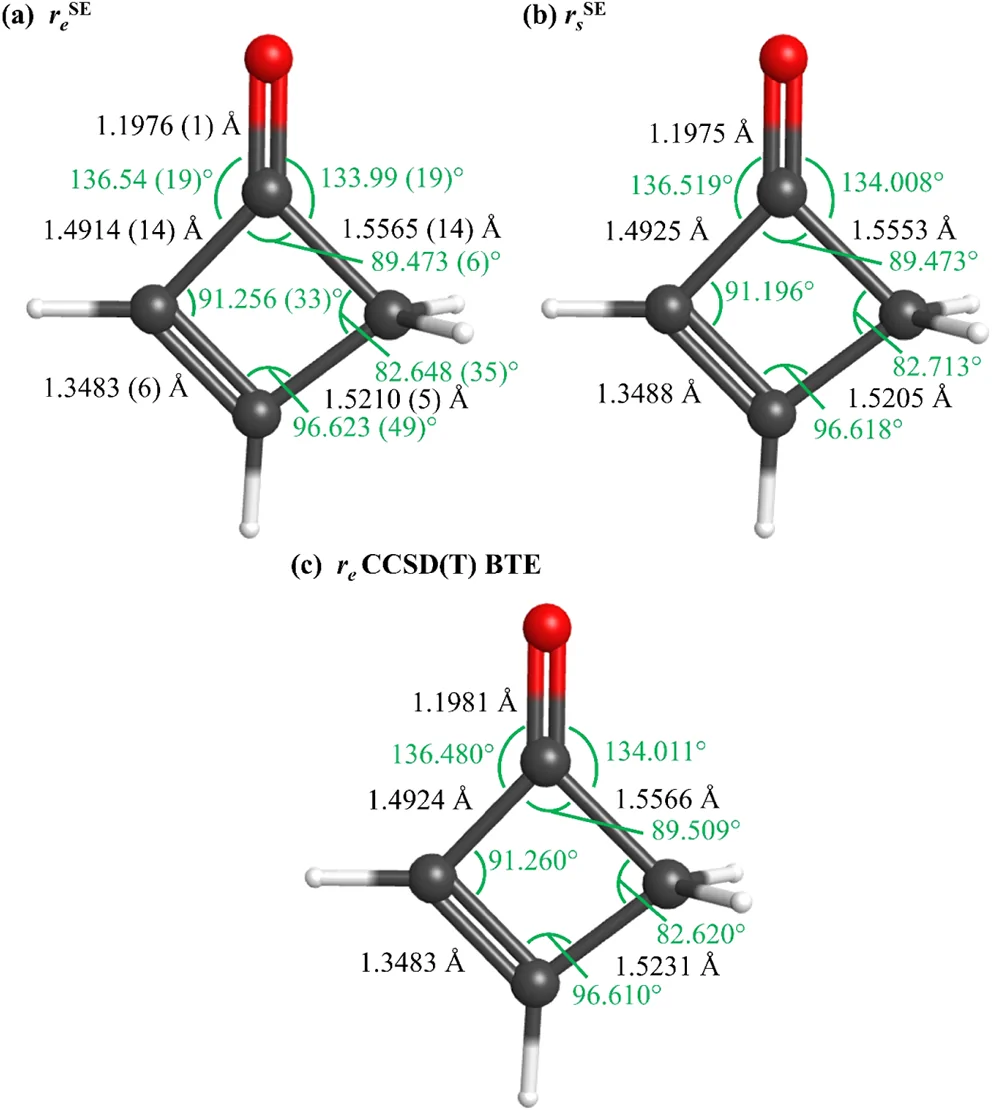
(a) Partial semi-experimental equilibrium (*r_e_* ^SE^), (b) partial semi-experimental substitution (*r_s_* ^SE^) structures of cyclobutenone, and (c) theoretical equilibrium structure (*r_e_* CCSD­(T) BTE). Uncertainties shown are 2σ and all corrections are calculated at the CCSD­(T)/cc-pCVTZ level.

The parameters of an *r*_*s*_^SE^ structure are determined by isotopic substitution of the atoms involved in that parameter and, in contrast to *r*_*e*_^SE^ structural parameters, are thus independent of any other structural parameters.^43,44^ The recent structural study of oxazole demonstrated that the same set of *B*_*e*_ constants provided very similar parameters in both the *r*_*e*_^SE^ and *r*_*s*_^SE^ structures,^45^ benchmarking an *r*_*s*_^SE^ structure with a very precise and accurate *r*_*e*_^SE^ structure for the first time. The small number of isotopologues available in this work motivated the comparison of the partial *r*_*e*_^SE^ and *r*_*s*_^SE^ structures of cyclobutenone. Based upon the comparison of the complete *r*_*s*_^SE^ and *r*_*e*_^SE^ structures for oxazole, the cyclobutenone *r*_*s*_^SE^ structural parameters are expected to be reliable to about 0.0010 Å and 0.04° for the bond distances and angles, respectively. The *r*_*s*_^SE^ parameters for cyclobutenone generally show reasonable agreement with the *r*_*e*_ BTE and *r*_*e*_^SE^ values at that level of assumed uncertainty. The notable exceptions are, once again, the *θ*_O–C1–C2_ and *θ*_O–C1–C4_ angles, where the near-axis locations of C1 and O result in *b*-coordinate values of −0.0237(51) and −0.0274(37), respectively. With only one reference geometry (the normal isotopologue) to complete Kraitchman’s analysis,^43,44^ no alternate values for these parameters can be determined. Thus, it is likely that the *r*_*s*_^SE^ geometry could be similarly improved by the incorporation of more isotopologues, particularly heavy-atom isotopologues with deuterium incorporation.

Despite having no isotopologues that involve ^2^H substitution, the *P*_*cc e*_ values provide perhaps the most reliable piece of structural information that can be obtained for cyclobutenone, *i*.*e*., the out-of-plane coordinate of the hydrogen atoms attached to the nominally *sp* ^3^-hybridized C4. As the only out-of-plane mass in cyclobutenone, the ^1^H atoms of each isotopologue reported in the current study are responsible for the non-zero value of *P*_*cc e*_. The distance of the ^1^H mass from the *ab*-plane, *c*_H_, is given by *c*_H_ = (*P*_*cc e*_/2*m*_H_)^1/2^ = [−1/2­(*I*_*c*_ – *I*_*a*_ – *I*_*b*_)/4*m*_H_]^1/2^.^15,31^ The values of *c*_H_ determined for each isotopologue are provided in Table 3 and result in an average semi-experimental value of 0.89218(7) Å, where the number in parentheses is the 2σ uncertainty. The remarkable similarity of the values for the [^13^C]-isotopologues and the differences to the normal and [^18^O]-isotopologues indicate the possibility of a systematic error introduced by the vibration-rotation interaction or electron-mass corrections. This assumption is supported by the lack of this pattern in the uncorrected, fully experimental *P*_*cc* 0_ values. It is possible that better corrections to the rotational constants could address this issue. The *r*_*e*_ *c*_H_ BTE value of 0.89176 Å is not in agreement with the semi-experimental value, indicating that improvement to either the computed corrections for the semi-experimental *P*_*cc* e_ values or improvements to the BTE structure may be necessary to improve the agreement.

### Lowest-Energy Vibrationally Excited State of Cyclobutenone: ν_21_

The lowest-energy vibrationally excited state of cyclobutenone (Figure 6) is the ring-puckering vibration (ν_21_, A″, 208.9 cm^–1^; CCSD­(T)/cc-pCVTZ). Over 3000 transitions for ν_21_ have been assigned, measured, and least-squares fit using an A-reduced sextic centrifugally distorted-rotor Hamiltonian in the I^*r*^ representation (σ_fit_ = 33 kHz). The resulting spectroscopic constants, which include a near-complete set of sextic centrifugal distortion constants, are provided in Table 5. The value of *Φ*_*K*_ was not determined in the least-squares fit and is held constant at its ground-state value. The quartic centrifugal distortion constants show the typical deviations from their corresponding ground-state values (2 to 18%)^46–49^ with the largest change observed for *Δ*_*K*_, which decreases by 18%. The sextic centrifugal distortion constants have discrepancies of 25% or less, also showing the expected changes relative to the ground state. The small changes of these constants support the treatment of ν_21_ with a single-state Hamiltonian, without explicit treatment of any coupling between states. The computed vibration-rotation interaction constants (*B*_0_ – *B*_21_) at the CCSD­(T)/cc-pCVTZ level (Table 6) are within 6% or 1.7 MHz of their experimental values. This excellent agreement both validates the computational method and provides additional evidence that ν_21_ is not impacted by any significant coupling interactions with neighboring vibrational states.

**Figure 6:**
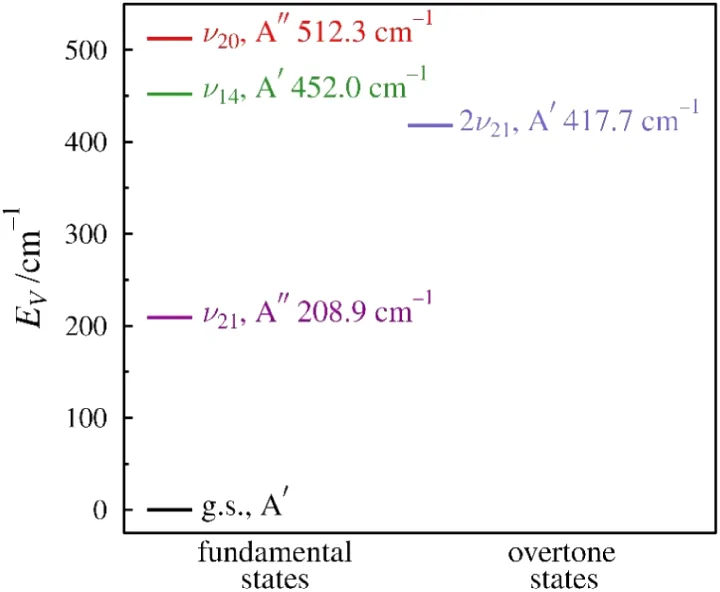
Vibrational energy levels of cyclobutenone below 520 cm^–1^ computed at the CCSD­(T)/cc-pCVTZ level of theory.

**Table 5:**
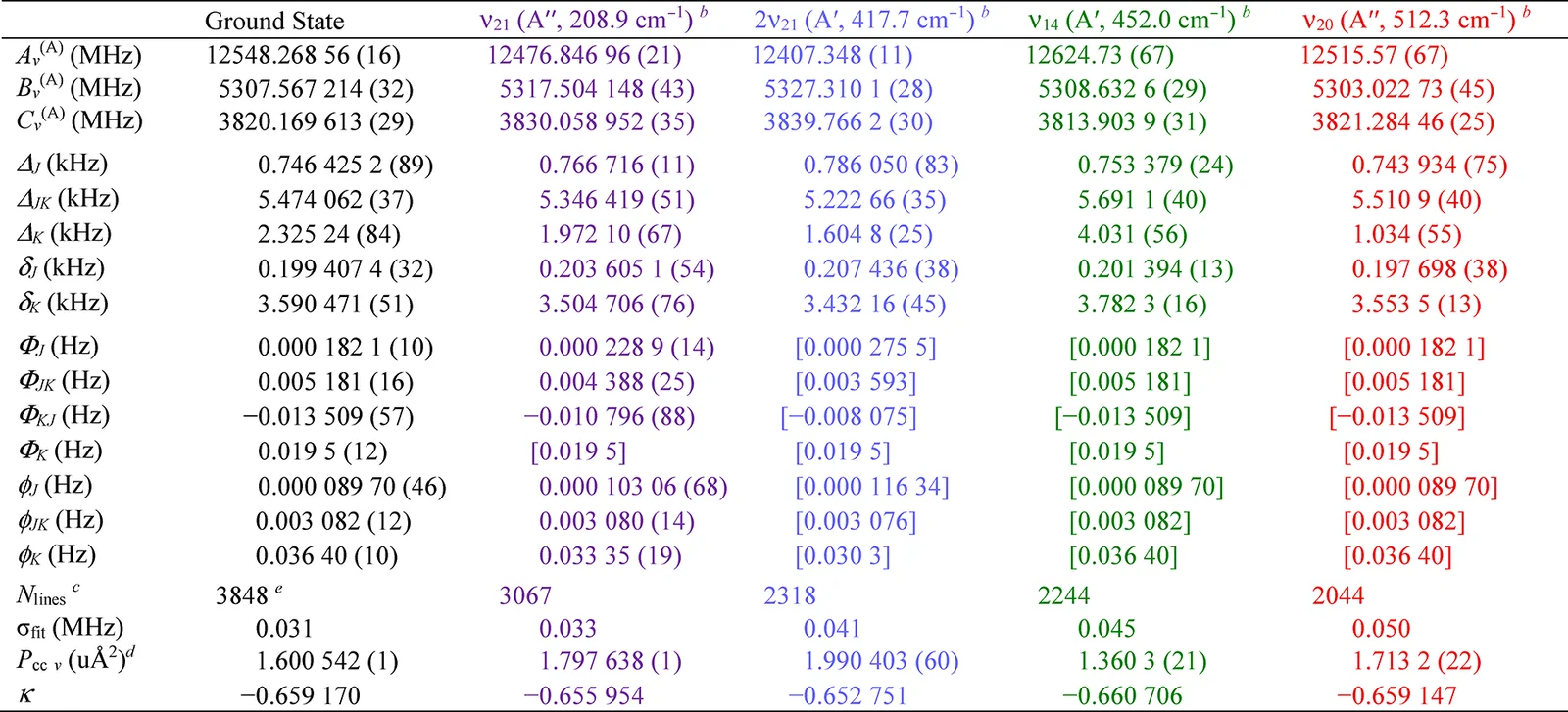
Experimental Spectroscopic Constants for Vibrationally Excited States ν_21_, 2ν_21_, ν_14_, and ν_20_ of Cyclobutenone (A-Reduced Hamiltonian, I*^r^* Representation)^a^ ^,^ ^b^ ^,^ ^c^ ^,^ ^d^ ^,^ ^e^

a Values in brackets held constant at the ground state (ν_21_, ν_14_, and ν_20_) or extrapolated value (2ν_21_).

b Energy calculated at the CCSD­(T)/cc-pCVTZ level.

c Number of independent transition frequencies.

d *P*_cc *v*_ = −(*I*_*c*_ – *I*_*a*_ – *I*_*b*_)/2 = −(1/2) *Δ*_*i*_ calculated from the *B*_*v*_ constants using PLANM.

e Ground-state transitions include those from previous work.^1^

**Table 6:**
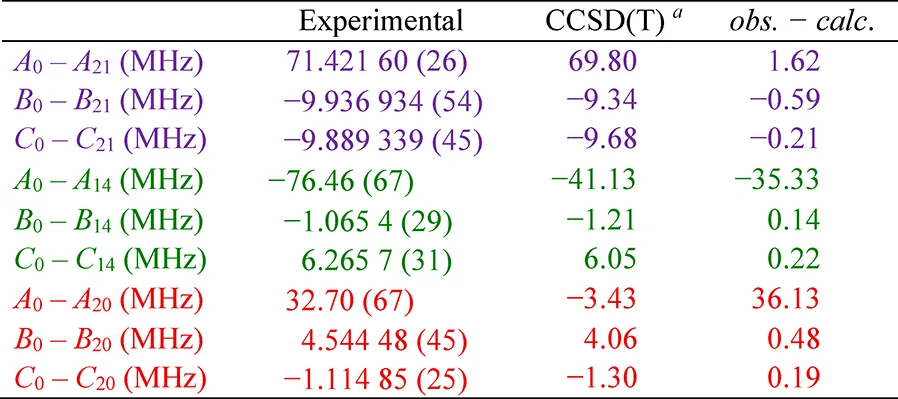
Experimental and Computed Vibration-Rotation Interaction Constants for Vibrationally Excited States ν_21_, ν_14_, and ν_20_ of Cyclobutenone^a^

a Evaluated with the cc-pCVTZ basis set.

### Low-Energy Fermi- and Coriolis-Coupled Triad of Cyclobutenone: 2ν_21_, ν_14_, and ν_20_

As shown in Figure 6, the next three lowest-energy vibrationally excited states of cyclobutenone are 2ν_21_, ν_14_, and ν_20_, which – due to their symmetries and vibrational energies – form a Coriolis- and anharmonic-coupled triad. As a result, a three-state Hamiltonian model was employed with the appropriate coupling terms. The *C*_*s*_ symmetry of cyclobutenone allows *a*- and *b*-axis coupling between states of different symmetry [2ν_21_ (A′) or ν_14_ (A′) with ν_20_ (A″)] and both *c*-axis and Fermi coupling between states of same symmetry [2ν_21_ (A′) and ν_14_ (A′)]. Over 2000 rotational transitions were measured, assigned, and least-squares fit for each of these states to an A-reduced, sextic centrifugally distorted Hamiltonian in the I^*r*^ representation with appropriate coupling terms.

The resulting spectroscopic constants for the three coupled states are displayed in Table 5 and Figure 7. The spectroscopic parameters that could not be determined for ν_14_ and ν_20_ were held constant at their ground-state values, while values for 2ν_21_ were extrapolated from the ground state and ν_21_. As a consequence of adding the coupling interaction terms between the states, the sextic centrifugal distortion constants could not be determined despite the large number of transitions measured for each state. The data distribution plots for these states are available in Figures S7–S10 and show only a slightly reduced range of *J*″ and *K*_*a*_″ values compared to the lower-energy states. Due to the need to use fewer centrifugal distortion terms and the difficulty in adequately modeling all of the coupling interactions, these states show a moderate increase in the σ_fit_ value. This behavior is similar to the increase in the σ_fit_ values as exemplified by the benzonitrile coupled dyads and triads relative to that of the ground-state value.^48,49^ Given the large *a*-axis Coriolis coupling between ν_14_ and ν_20_, it is unsurprising that the *A*_0_ *– A*_14_ and *A*_0_ *– A*_20_ values show large, nearly equal but opposite deviations from their computationally predicted values of ±36 MHz (Table 6). CFOUR does not deperturb the impact of Coriolis coupling on the *B*_0_ *– B*_*v*_ values, and it is very difficult to ascertain whether the experimental values have been fully deperturbed by adequate treatment of the coupling interations. The smaller remaining *a*-, *b*- and *c*-axis couplings do not have a similar impact on the other vibration-rotation interaction contants, as shown by the small *obs. – calc*. values < 1.6 MHz (Table 6). This close agreement of the remaining vibration-rotation interaction constants validates both the computed and experimentally determined *B*_*v*_ and *C*_*v*_ values. Importantly, all of the determined quartic centrifugal distortion constants for the fundamental states are similar to the corresponding values of ν_21_ and the ground state (Table 5), indicating that they are predominantly serving their intended function of modeling centrifugal distortion, while the Coriolis and Fermi couplings are being mainly addressed by their respective terms (as they should be). Possible exceptions to the previous statement are the *Δ*_*K*_ values of ν_20_ and ν_14_, which show larger deviations than typical. They do not, however, display the equal and opposite deviations typical for distortion constants that have absorbed Coriolis coupling.^48–51^

**Figure 7:**
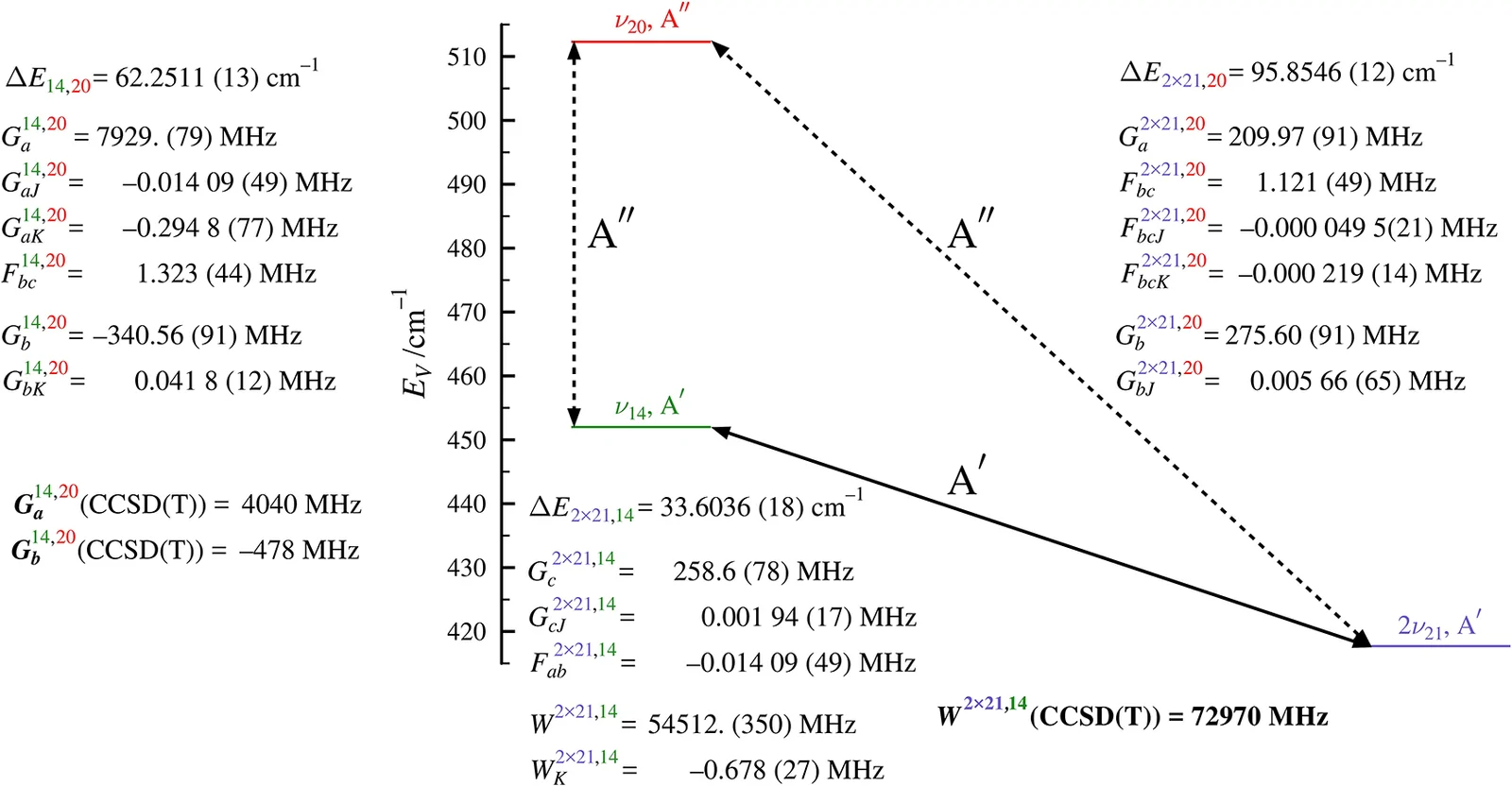
Coupling coefficients for the 2ν_21_ (A′), ν_14_ (A′), and ν_20_ (A″) Coriolis and anharmonic interactions for cyclobutenone obtained from the least-squares fit of the triad. Symmetry terms adjacent to arrows indicate the symmetry of the corresponding interaction.

While the lack of direct computational comparison makes it difficult to discern whether coupling has been effectively eliminated from the *A*_*v*_ constants of the fundamental states, greater confidence can be found examining the values determined for 2ν_21_. The determined value of *A* is less than 2 MHz (0.02%) from the value extrapolated from the ground and first fundamental states. In fact, all of the determined constants for the overtone state are less than 1% different from their extrapolated values, validating both the lower-energy state determinations and the successful addressing of coupling in 2ν_21_. Taken together, the data indicate that the constants have been meaningfully determined across the coupled excited states, which provides some confidence that the spectroscopic constants from this least-squares fit have physical meaning and can serve as reasonable computational benchmarks.

With no available high-resolution infrared spectra available for cyclobutenone, the precise and accurate absolute energies of these vibrational states are unknown. The coupling interactions and inclusion of strongly perturbed transitions in the data set for the triad, however, determine precise and accurate energy separations between the vibrationally excited states (*E*_20,2×21_ = 95.8546(12) cm^–1^, *E*_20,14_ = 62.2511(13) cm^–1^, and *E*_2×21,14_ = 33.6036(18) cm^–1^). Several combinations of Coriolis and anharmonic coupling constants were explored to obtain a set of them that could adequately model the data set. While it is likely that other combinations of coupling constants could be used to obtain satisfactory least-squares fits, the energies between the states are likely to be the most physically meaningful values obtained from the least-squares fit. The value of *E*_20,14_ is particularly reliable because those states interact by anharmonic (Fermi) coupling. *A priori* computational prediction of many of the Coriolis and anharmonic coupling constants determined in this work remains a challenge as appropriate methodology is not implemented in current computational software (in particular, for overtone or combination states). The Coriolis zeta constants $(\zeta_{a}^{20,14}=-0.161$ and $\zeta_{b}^{20,14}=-0.045$ ; CCSD­(T)/cc-pCVTZ), are provided in the anharmonic frequency calculation and used to estimate $G_{a}^{20,14}$ and $G_{b}^{20,14}$ by the relation that $G_{b}^{20,14}\approx 2B\zeta_{b}^{20,14}$ . While the predicted value of $G_{b}^{20,14}$ (−478 MHz) is plausibly close to the determined value (−341 MHz), the predicted value of $G_{a}^{20,14}$ (4040 MHz) is only half of the determined value of 7929 MHz. The perturbation analysis that generates the $\zeta_{a}^{20,14}$ value assumes a two-state interaction between ν_14_ and ν_20_ that can be fully modeled by a single term. In a triad of states, the energy levels can be impacted by mixing of the third state (2ν_21_) of the triad. While this is a potential issue, the agreement between the computed and experimental ζ values for the analogous coupled triad of glycolic acid is better than those presented here for cyclobutenone, even with the glycolic acid values calculated at a lower level of theory.^52^ The origin of this discrepancy between the computed and experimental $\zeta_{a}^{20,14}$ and $\zeta_{b}^{20,14}$ values is not clear and may indicate that these values are too highly correlated in the least-squares fit to the other coupling terms between ν_20_ and ν_14_. The anharmonic coupling (*W*), estimated to be *k*_21,21,14_/4, is 72,970 MHz at the CCSD­(T) level. Similarly to the *G*_*x*_ terms, there is a significant disagreement between the computed and experimental values (Figure 7). Despite the possibility that the values of *W* ^20,14^, $\zeta_{a}^{20,14}$ , and $\zeta_{b}^{20,14}$ are somewhat effective, rather than physically meaningful, the combination of Coriolis and Fermi-coupling constants do an excellent job of addressing the interaction between these states. The rotational energy levels computed from these spectroscopic constants reproduce many highly perturbed transition frequencies (resonant transitions and coupling-allowed, nominal interstate transitions) within 3σ.

The coupling-induced state mixing that occurs between 2ν_21_, ν_14_, and ν_20_ causes many of the *K*_*a*_ series for each state to experience mild perturbation, resulting in a curvature that the centrifugal distortion terms alone cannot accommodate. Where the rotational energy levels cross, the perturbation is much greater, causing the sharp resonances in the *K*_*a*_ series shown in Figure 8. While assigning and fitting the transitions involved in these sharp resonances may be challenging, they provide precise and accurate determination of the energy separations. In Figure 8, moderately sized resonances are shown between 2ν_21_ *K*_*a*_ = 6^+^ with ν_14_ *K*_*a*_ = 5^–^ at *J*″ + 1 = 71–73. Also featured is a set of smaller resonances between ν_14_ *K*_*a*_ = 11^–^ with ν_20_ *K*_*a*_ = 5^+^ at *J*″ + 1 = 64 and 65. The numerous measured resonances enable fairly precise determination of the coupling constants. Figure 9 presents a resonance progression plot for the even *K*_*a*_^+^ series of ν_14_, showcasing the evolution of the resonances over *J* and *K*_*a*_. The observed transitions with most extreme perturbations were displaced from their unperturbed frequencies by more than 630 MHz. Transitions with more extreme perturbations were too weak to be observed, due to intensity borrowing from their corresponding nominal interstate transitions.

**Figure 8:**
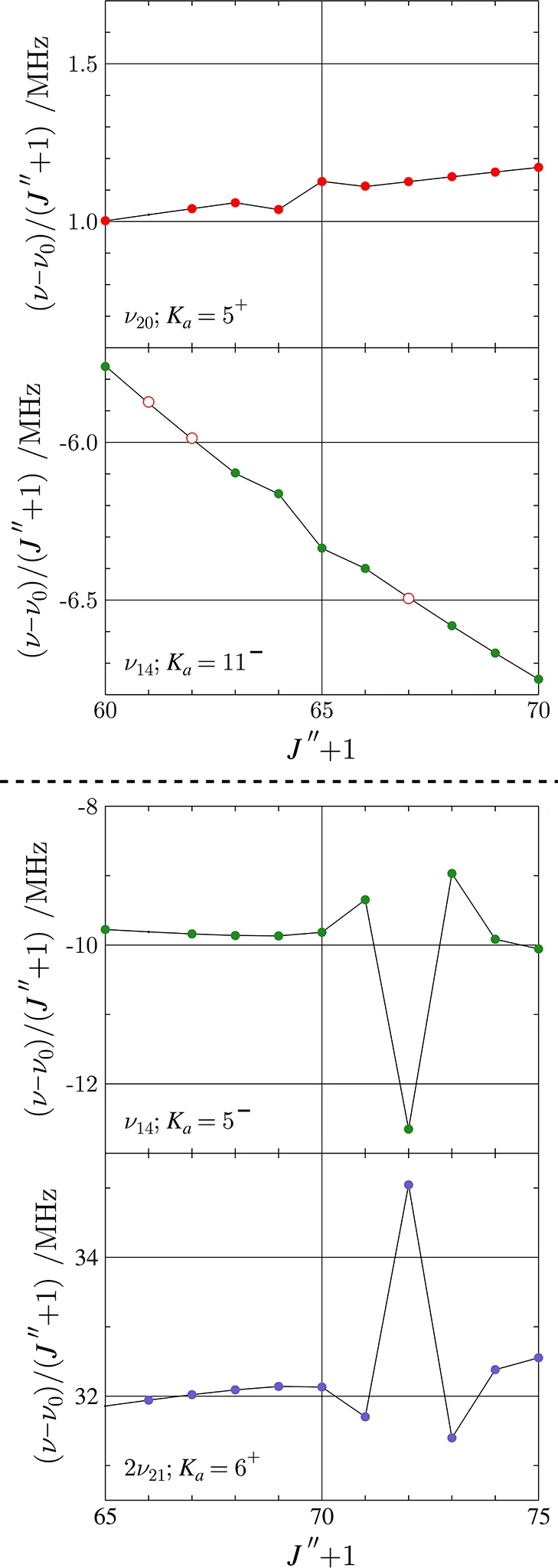
Resonance plots for cyclobutenone showing the *K_a_* = 6^+^ series (slate blue) for 2ν_21_ and the *K_a_* = 5^–^ series (green) for ν_14_ (bottom), as well as the *K_a_* = 11^–^ series (green) for ν_14_ and the *K_a_* = 5^+^ series (red) for ν_20_ (top), representing the *b*-axis Coriolis interaction of the two lower-energy states and the *a*-axis Coriolis interaction of the two higher-energy states. Only the *K_a_* = 11^–^ series (green) for ν_14_ contains transitions for which the *obs. – calc*. value exceeds 150 kHz or 3 times the nominal experimental uncertainty (open red circles). The plotted values are frequency differences between excited-state transitions and their ground-state counterparts (ν – ν_0_), scaled by (*J″* + 1) in order to make the plots more horizontal. Predictions from the final coupled fit are represented by a solid, black line.

**Figure 9:**
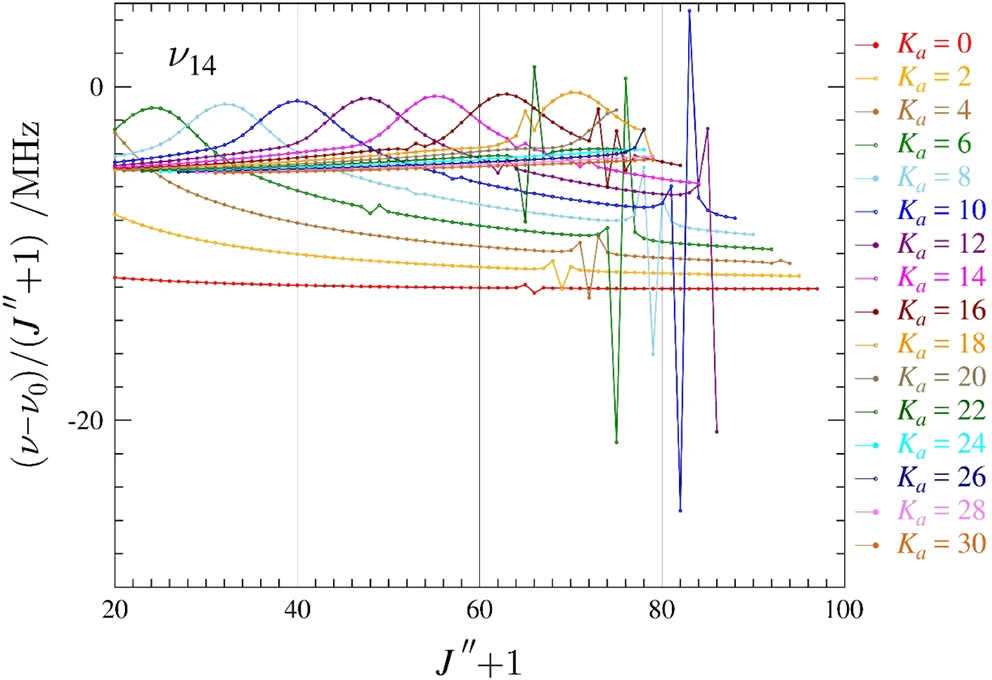
Superimposed resonance plots of ν_14_ for *^a^* R_0,1_ even-*K_a_* ^+^ series from 0 to 30 for cyclobutenone. Measured transitions are omitted for clarity, but they are indistinguishable from the plotted values on this scale. The plotted values are frequency differences between excited-state transitions and their ground-state counterparts (ν – ν_0_), scaled by (*J*″ + 1).

The intense state-mixing that causes the displaced resonant transitions shown in Figures 8 and 9 results in observable, formally forbidden, nominal interstate transitions. Signal intensity is transferred from the resonant intrastate transitions to formally interstate transitions that change both rotational and vibrational quanta. Consequently, 11 independent transition frequencies of nominal interstate transitions were observed between 2ν_21_ and ν_14_, which are separated by 33.6036(18) cm^–1^. An example of a matched pair of intra- and interstate transitions is highlighted in Figure 10. The measurement of a few such sets provides absolute confidence in their assignment. As they share the same rotational energy levels, the difference between the average of the interstate transition frequencies (*x̅*_*INTER*_) and the average of the intrastate transition frequencies (*x̅*_*INTRA*_) should be identical within the measurement uncertainty (50 kHz for each transition or 71 kHz for their average). In the case shown, the transition frequency averages differ by less than 7 kHz. In order for the model to predict and fit these highly perturbed intrastate transitions and coupling-allowed nominal interstate transitions, the energy separations between the states must be very well modeled.

**Figure 10:**
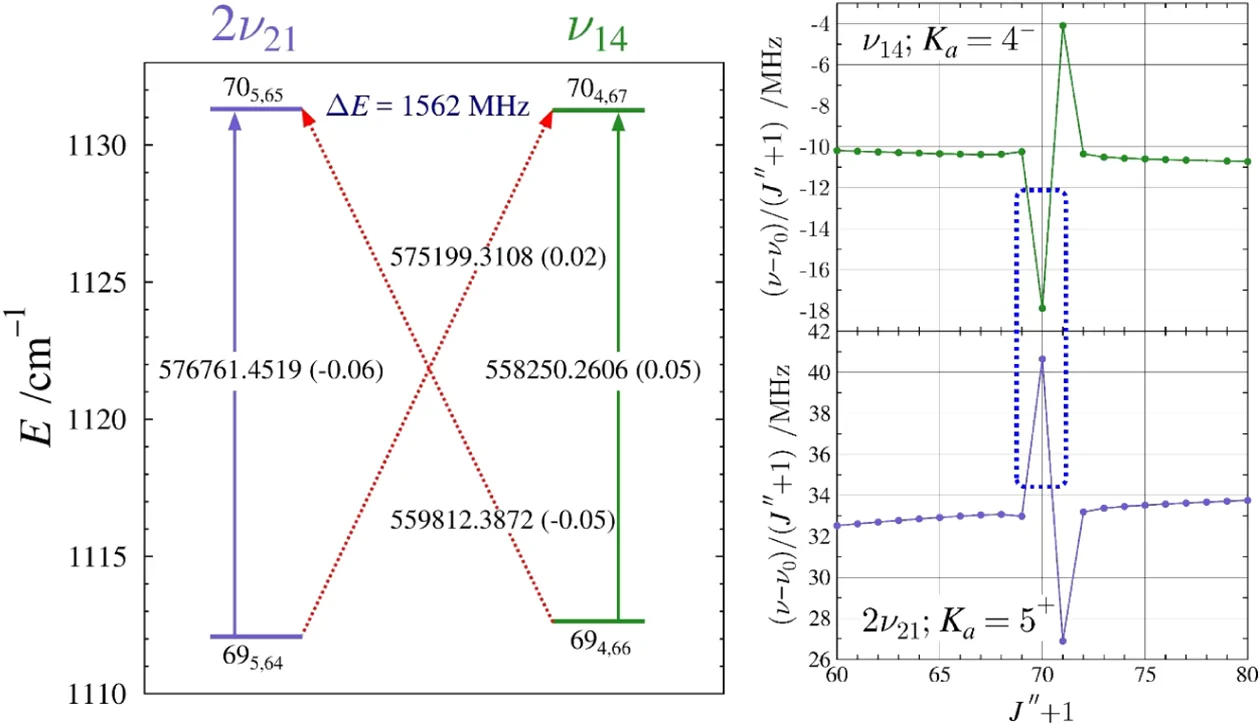
Energy diagram (left) depicting a representative matched pair of nominal interstate transitions between the 2ν_21_ (slate blue) and ν_14_ (green) vibrationally excited states of cyclobutenone. Standard *^a^* R_0,1_ transitions within vibrational states are denoted by vertical arrows. The diagonal, dashed arrows indicate nominal interstate transitions. The values printed on each of the arrows are the corresponding transition frequency (in MHz) with the corresponding *obs*. – *calc*. value in parentheses. The marked energy separation is between the two strongly interacting rotational energy levels. Resonance plot (right) of the *K_a_* series of the corresponding resonant intrastate transitions.

## Conclusion

The rotational spectrum of cyclobutenone has been studied from 9 to 735 GHz, providing a complete set of spectroscopic parameters for a sextic centrifugally distorted Hamiltonian and the spectroscopic data needed for future radioastronomical searches. Partial semi-experimental equilibrium (*r*_*e*_^SE^) and substitution (*r*_*s*_^SE^) structures have been determined for this highly strained four-membered ring via analysis of the spectra of its [^13^C]- and [^18^O]-isotopologues. While the structural parameters provide insight into the shape of the strained ring, it is clear that improvements to the *r*_*e*_^SE^ and *r*_*s*_^SE^ structures are possible with syntheses and spectroscopic study of deuterium-substituted isotopologues. While a satisfactory partial *r*_*e*_^SE^ structure was obtained, the larger-than-expected differences between the *r*_*e*_^SE^ and *r*_*e*_ BTE parameters highlight the need for all atoms to be substituted in order to achieve agreement between theory and experiment. The *r*_*s*_^SE^ structure could also be improved by obtaining additional reference structures without the C1 and O atoms near the *a*-axis. Importantly, the *r*_*e*_^SE^ structural analysis of cyclobutenone validates the methodology to obtain highly accurate structural parameters by improving the CCSD­(T)/cc-pCV5Z parameter values with additional corrections addressing finite basis set, higher-level electron correlation, and relativistic effects, as well as the diagonal Born–Oppenheimer correction. Analysis of the vibrationally excited states up to 520 cm^–1^ provides spectroscopic constants for vibrationally excited states ν_21_, 2ν_21_, ν_14_, and ν_20_ and the precise and accurate energy separations between 2ν_21_, ν_14_, and ν_20_. These values may be used for future computational benchmarking.

## Supplementary Material





## Notes

Equations used to calculate determinable constants, equations and corrections used to calculate the BTE, A- and S-reduction spectroscopic constants, data distribution plots, experimental details for synthesis and characterization of cyclobutenone (PDF)
Least-squares fitting output files, computational output files, xrefit output files (ZIP)

## References

[1] Carrillo M. J., Marasinghe D., Feeley E. B., Sobie K. M., Zarzycki R. J., Carter-Fenk K., Fenk C. J., Tubergen M. J. (2023). Theoretical and
Microwave Spectroscopic Characterization of Cyclobutenone: Planar
or Puckered?. J. Phys. Chem. A.

[2] Etim E. E., Godwin O. E., Sambo I. F., Olagboye S. A. (2020). Quantum chemical
studies on furan and its isomers. Int. J. Mod.
Chem..

[3] Dutta S., Lu Y.-L., Erchinger J. E., Shao H., Studer E., Schäfer F., Wang H., Rana D., Daniliuc C. G., Houk K. N., Glorius F. (2024). Double Strain-Release [2π+2σ]-Photocycloaddition. J. Am. Chem. Soc..

[4] Bauzá A., Quiñonero D., Deyà P. M., Frontera A. (2012). Estimating ring strain
energies in small carbocycles by means of the Bader’s theory
of ‘atoms-in-molecules’. Chem.
Phys. Lett..

[5] Niwayama S., Kallel E. A., Sheu C., Houk K. N. (1996). Theoretical Predictions
of Substituent Effects on the Thermal Electrocyclic Ring Openings
of Cyclobutenones. J. Org. Chem..

[6] McAllister M. A., Tidwell T. T. (1994). *Ab Initio* Calculations of the Ring
Opening of Cyclobutene-1,2-diones and Conformational Properties of
the Product 1,3-Butadiene-1,4-diones (Bisketenes). J. Am. Chem. Soc..

[7] Cope A. C., Schaeren S. F., Trumbull E. R. (1954). Cyclic Polyolefins. XXX. Reactions
of Cycloöctatrienone; Ethoxycycloöctatetraene. J. Am. Chem. Soc..

[8] Vogel E., Hasse K. (1958). Kleine Kohlenstoff-Ringe III. Versuche zur Synthese von Cyclobutenon. Justus Liebigs Ann. Chem..

[9] Sieja J. B. (1971). Synthesis
of cyclobutenone. J. Am. Chem. Soc..

[10] Ross A. G., Li X., Danishefsky S. J. (2012). Preparation
of cyclobutenone. Org. Synth..

[11] Li X., Danishefsky S. J. (2010). Cyclobutenone as a Highly Reactive
Dienophile: Expanding
Upon Diels–Alder Paradigms. J. Am. Chem.
Soc..

[12] Ding W.-J., Fang D.-C. (1999). DFT and topological
study on the mechanism of ring
opening of cyclobutenone, azetinone and oxetone. J. Mol. Struct. Theochem..

[13] Kikuchi O. (1982). An MO Study
of the Photochemical Electrocyclic Isomerization between Vinylketene
and Cyclobutenone. Bull. Chem. Soc. Jpn..

[14] The Cologne Database for Molecular Spectroscopy; https://cdms.astro.uni-koeln.de/classic/molecules (accessed 22-July-2026).

[15] Smith H. H., Esselman B. J., Wood S. A., Stanton J. F., Woods R. C., McMahon R. J. (2023). Improved Semi-Experimental
Equilibrium Structure and
High-Level Theoretical Structures of Ketene. J. Chem. Phys..

[16] Conrad A. R., Teumelsan N. H., Wang P. E., Tubergen M. J. (2010). A Spectroscopic
and Computational Investigation of the Conformational Structural Changes
Induced by Hydrogen Bonding Networks in the Glycidol–Water
Complex. J. Phys. Chem. A.

[17] Pickett H. M. (1980). Determination
of Collisional Linewidths and Shifts by a Convolution Method. Appl. Opt..

[18] Kisiel Z., Pszczółkowski L., Drouin B. J., Brauer C. S., Yu S., Pearson J. C., Medvedev I. R., Fortman S., Neese C. (2012). Broadband
Rotational Spectroscopy of Acrylonitrile: Vibrational Energies from
Perturbations. J. Mol. Spectrosc..

[19] Kisiel Z., Pszczółkowski L., Medvedev I. R., Winnewisser M., De Lucia F. C., Herbst E. (2005). Rotational
Spectrum of *trans–trans* Diethyl Ether in the
Ground and Three Excited Vibrational States. J. Mol. Spectrosc..

[20] Pickett H. M. (1991). The Fitting
and Prediction of Vibration-Rotation Spectra with Spin Interactions. J. Mol. Spectrosc..

[21] Kisiel, Z. , PROSPE - Programs for Rotational SPEctroscopy; http://info.ifpan.edu.pl/~kisiel/prospe.htm (accessed 8-July-2026).

[22] Heim Z. N., Amberger B. K., Esselman B. J., Stanton J. F., Woods R. C., McMahon R. J. (2020). Molecular Structure Determination:
Equilibrium Structure
of Pyrimidine (*m*-C_4_H_4_N_2_) from Rotational Spectroscopy (*r_e_
*
^SE^) and High-Level *ab Initio* Calculation
(*r_e_
*) Agree Within the Uncertainty of Experimental
Measurement. J. Chem. Phys..

[23] Esselman B. J., Zdanovskaia M. A., Owen A. N., Stanton J. F., Woods R. C., McMahon R. J. (2021). Precise Equilibrium Structure of Thiazole (*c*-C_3_H_3_NS) from Twenty-Four Isotopologues. J. Chem. Phys..

[24] Owen A. N., Zdanovskaia M. A., Esselman B. J., Stanton J. F., Woods R. C., McMahon R. J. (2021). Semi-Experimental
Equilibrium (*r_e_
*
^SE^) and Theoretical
Structures of Pyridazine
(*o*-C_4_H_4_N_2_). J. Phys. Chem. A.

[25] Bunn H. A., Esselman B. J., Franke P. R., Kougias S. M., McMahon R. J., Stanton J. F., Widicus Weaver S. L., Woods R. C. (2022). Millimeter/Submillimeter-wave
Spectroscopy and the Semi-Experimental Equilibrium (*r_e_
*
^SE^) Structure of 1*H*-1,2,4-Triazole
(*c*-C_2_H_3_N_3_). J. Phys. Chem. A.

[26] Owen A. N., Sahoo N. P., Esselman B. J., Stanton J. F., Woods R. C., McMahon R. J. (2022). Semi-Experimental Equilibrium (*r_e_
*
^SE^) and Theoretical Structures of
Hydrazoic Acid
(HN_3_). J. Chem. Phys.

[27] Zdanovskaia M. A., Esselman B. J., Kougias S. M., Amberger B. K., Stanton J. F., Woods R. C., McMahon R. J. (2022). Precise Equilibrium Structures of
1*H*- and 2*H*-1,2,3-Triazoles (C_2_H_3_N_3_) by Millimeter-Wave Spectroscopy. J. Chem. Phys..

[28] Esselman B.
J., Zdanovskaia M. A., Owen A. N., Stanton J. F., Woods R. C., McMahon R. J. (2023). Precise
Equilibrium Structure of Benzene. J. Am. Chem.
Soc..

[29] Zdanovskaia M. A., Esselman B. J., Kougias S. M., Atwood M. G., Jones G. H., Stanton J. F., Woods R. C., McMahon R. J. (2025). Precise Semi-Experimental
Equilibrium (*r_e_
*
^SE^) Structure
of Pyridine from 32 Isotopologues: Accurate Assessment of the Effect
of Nitrogen-Atom Substitution in Aromatic Rings. J. Phys. Chem. A.

[30] Esselman B. J., Atwood M. G., Nguyen H. V. L., Stanton J. F., Woods R. C., McMahon R. J. (2025). Semi-experimental equilibrium structure
(*r_e_
*
^SE^) of thiophene: Attempting
to resolve
the heavy-atom problem with 46 isotopologues. J. Chem. Phys..

[31] Styers W. H., Esselman B. J., Wood S. A., Changala P. B., Jones G. H., McCarthy M. C., Stanton J. F., Woods R. C., McMahon R. J. (2026). Cyclopropanone:
Preparation, Rotational Spectroscopy, and Semi-Experimental Equilibrium
(*r*
_
*e*
_
^SE^) Structure. J. Am. Chem. Soc..

[32] Stanton, J. F. ; Gauss, J. ; Cheng, M. E. ; Harding, M. E. ; Matthews, D. A. ; Szalay, P. G. ; Asthana, A. ; Auer, A. A. ; Bartlett, R. J. ; Benedikt, U. ; Berger, C. ; Bernholdt, D. E. ; Blaschke, S. ; Bomble, Y. J. ; Christiansen, O. ; Datta, D. ; Engel, F. ; Faber, R. ; Greiner, J. ; Heckert, M. ; Heun, O. ; Hilgenberg, M. ; Huber, C. ; Jagau, T.-C. ; Jonsson, D. ; Jusélius, J. ; Kirsch, T. ; Kitsaras, M.-P. ; Klein, K. ; Kopper, G. M. ; Lauderdale, W. J. ; Lipparini, F. ; Liu, J. ; Metzroth; Monzel, L. ; Mück, L. A. ; Nottoli, T. ; O’Neill, D. P. ; Oswald, J. ; Price, D. R. ; Prochnow, E. ; Puzzarini, C. ; Ruud, K. ; Schiffmann, F. ; Schwalbach, W. ; Simmons, C. ; Stopkowicz, S. ; Tajti, A. ; Uhlířová, T. ; Vázquez, J. ; Wang, F. ; Watts, J. D. ; Yergün, P. ; Zhang, C. ; Zheng, X. ; Almlöf, J. ; Taylor, P. R. ; Helgaker, T. ; Jensen, H. J. A. ; Jørgensen, P. ; Olsen, J. ; Mitin, A. V. ; van Wüllen, C. ; CFOUR, Coupled-Cluster Techniques for Computational Chemistry. For the current version, see http://www.cfour.de (accessed 21-Jul-2026).

[33] Peterson K. A., Dunning T. H. (2002). Accurate correlation
consistent basis
sets for molecular core–valence correlation effects: The second
row atoms Al–Ar, and the first row atoms B–Ne revisited. J. Chem. Phys..

[34] Dunning T. H. (1989). Gaussian basis sets for use in correlated molecular
calculations. I. The atoms boron through neon and hydrogen. J. Chem. Phys..

[35] Kendall R. A., Dunning T. H., Harrison R. J. (1992). Electron affinities
of the first-row atoms revisited. Systematic basis sets and wave functions. J. Chem. Phys..

[36] Morgan W. J., Matthews D. A., Ringholm M., Agarwal J., Gong J. Z., Ruud K., Allen W. D., Stanton J. F., Schaefer H. F. (2018). Geometric Energy Derivatives at the Complete Basis
Set Limit: Application to the Equilibrium Structure and Molecular
Force Field of Formaldehyde. J. Chem. Theory
Comput..

[37] Puzzarini C., Bloino J., Tasinato N., Barone V. (2019). Accuracy and Interpretability:
The Devil and the Holy Grail. New Routes across Old Boundaries in
Computational Spectroscopy. Chem. Rev..

[38] Gauss J., Puzzarini C. (2010). Quantum-chemical
calculation of Born-Oppenheimer breakdown
parameters to rotational constants. Mol. Phys..

[39] Mills, I. M. Vibration-Rotation Structure in Asymmetric- and Symmetric-Top Molecules. In Molecular Spectroscopy: Modern Research; Rao, K. N. ; Mathews, C. W. , Eds.; Academic Press: New York, 1972; Vol.: 1, pp. 115–140.

[40] Schneider W., Thiel W. (1989). Anharmonic Force Fields
from Analytic Second Derivatives: Method
and Application to Methyl Bromide. Chem. Phys.
Lett..

[41] Stanton J. F., Lopreore C. L., Gauss J. (1998). The equilibrium structure and fundamental
vibrational frequencies of dioxirane. J. Chem.
Phys..

[42] Gordy, W. ; Cook, R. Microwave Molecular Spectra; 3rd ed.; Wiley Interscience: New York, 1984.

[43] Kraitchman J. (1953). Determination
of Molecular Structure from Microwave Spectroscopic Data. Am. J. Phys..

[44] Rudolph H. D. (1981). Extending
Kraitchman’s equations. J. Mol. Spectrosc..

[45] Esselman B. J., Zdanovskaia M. A., Atwood M. G., Adkins T. K., Kobayashi M., Tsunekawa S., Kobayashi K., Sahoo N. P., Stanton J. F., Woods R. C., McMahon R. J. (2026). Improved Accuracy in Semi-Experimental
Structure Determination by Resolving Problems Associated with Rotation
of Principal Inertial Axes of Isotopologues: Structures of 1,3-Oxazole
(*c*-C_3_H_3_NO). J. Phys. Chem. A.

[46] Watson J. K. G. (2005). The
vibrational dependence of quartic centrifugal distortion. J. Mol. Struct..

[47] Zdanovskaia M. A., Franke P. R., Esselman B. J., Billinghurst B. E., Zhao J., Stanton J. F., Woods R. C., McMahon R. J. (2023). Vibrationally
excited states of 1*H*- and 2*H*-1,2,3-triazole
isotopologues analyzed by millimeter-wave and high-resolution infrared
spectroscopy with approximate state-specific quartic distortion constants. J. Chem. Phys..

[48] Zdanovskaia M. A., Esselman B. J., Lau H. S., Bates D. M., Woods R. C., McMahon R. J., Kisiel Z. (2018). The 103 – 360 GHz Rotational
Spectrum of Benzonitrile, the First Interstellar Benzene Derivative
Detected by Radioastronomy. J. Mol. Spectrosc..

[49] Zdanovskaia M. A., Martin-Drumel M.-A., Kisiel Z., Pirali O., Esselman B. J., Woods R. C., McMahon R. J. (2022). The eight lowest-energy vibrational
states of benzonitrile: analysis of Coriolis and Darling-Dennison
couplings by millimeter-wave and far-infrared spectroscopy. J. Mol. Spectrosc..

[50] Sarka K. (1972). Anomalous
values of the rotational constants of the parallel bands perturbed
by Coriolis resonance. J. Mol. Spectrosc..

[51] Christopher D. T., Evan G. R., McNaughton D. (2004). Completing
the picture in the rovibrational
analysis of chlorodifluoromethane (CHClF_2_): ν_3_ and ν_8_. Mol. Phys..

[52] Kisiel Z., Pszczółkowski L., Białkowska-Jaworska E., Charnley S. B. (2016). Millimetre wave
rotational spectrum of glycolic acid. J. Mol.
Spectrosc..

