# Supplementary material for: Rotational Spectroscopy and Partial Semi-Experimental Equilibrium (re SE) and Substitution (rs SE) Structures of Cyclobutenone (c‑C4H4O)
Source: J Phys Chem A. 2026 Sep 28;130(40):7953–65. doi: 10.1021/acs.jpca.6c04937 (PMC13656784; doi:10.1021/acs.jpca.6c04937)
Supplement: Supplementary file 1 [file jp6c04937_si_001.pdf]

Supporting Information

Rotational Spectroscopy and Partial Semi-Experimental Equilibrium (r_e^{SE}) and Substitution (r_s^{SE}) Structures of Cyclobutenone (c-C_4H_4O)

Brian J. Esselman,[1] Michael J. Carrillo,[2] Madeleine G. Atwood,[1]

Nitai P. Sahoo,[3] Zoe K. Deems,[2] Michael J. Tubergen,[2]

Maria A. Zdanovskaia,[1] R. Claude Woods,[1] Robert J. McMahon[1,]*

[1] *Department of Chemistry, University of Wisconsin–Madison, Madison, Wisconsin 53706, United States*

[2] *Department of Chemistry and Biochemistry, Kent State University, Kent, Ohio 44240, United States*

[3] *Quantum Theory Project, Departments of Physics and Chemistry, University of Florida, Gainesville, Florida 32611, United States*

* corresponding author

E-mail address: robert.mcmahon@wisc.edu (R.J. McMahon)

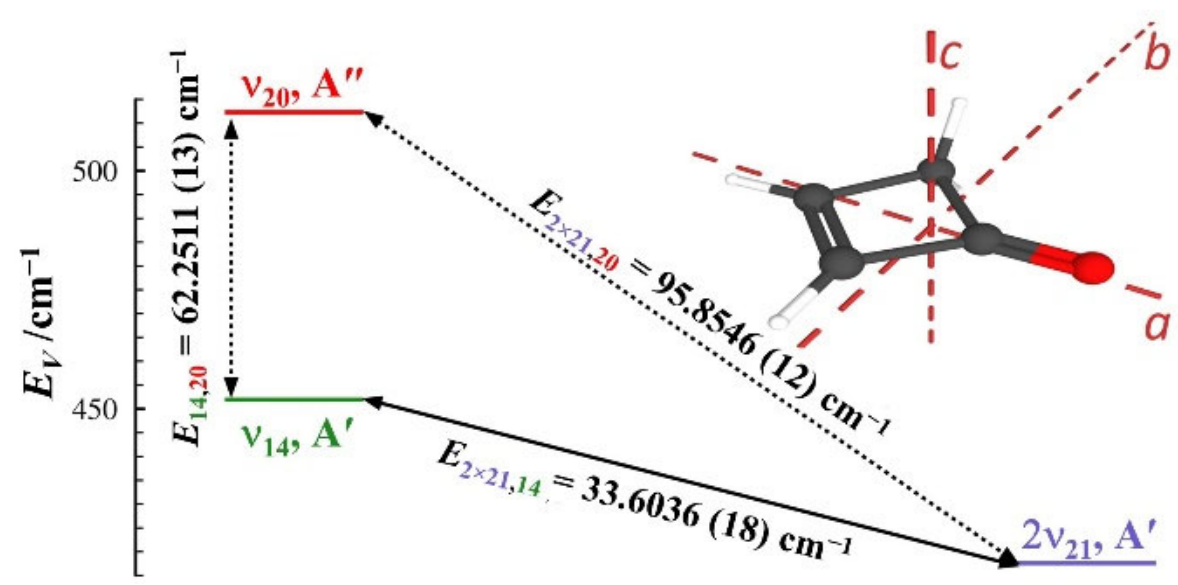

Table of Contents		Page
**Eq. S1 – S6**.	Calculating determinable constants	S4
**Eq. S7**	Calculating equilibrium rotational constant	S4
**Eq. S8 – S14.**	Calculating the Best Theoretical Estimate (BTE)	S4
**Table S1.**	Corrections (ΔR) to CCSD(T)/cc-pCV5Z used to obtain the Best Theoretical Estimate (BTE)	S6
	**Isotopologue Numbering**	S6
	**Overview of tables of spectroscopic constants and figures of data distribution plots**	S6
**Table S2.**	Experimental and computed spectroscopic constants for the normal isotopologue of cyclobutenone.	S7
**Figure S1.**	Data distribution plot for the least-squares fit of millimeter-wave spectroscopic data for the normal isotopologue of cyclobutenone	S8
**Table S3**.	Experimental and computed spectroscopic constants for [1-^{13}C]-cyclobutenone	S9
**Figure S2.**	Data distribution plot for the least-squares fit of millimeter-wave spectroscopic data for [1-^{13}C]-cyclobutenone	S10
**Table S4**.	Experimental and computed spectroscopic constants for [2-^{13}C]-cyclobutenone	S11
**Figure S3.**	Data distribution plot for the least-squares fit of millimeter-wave spectroscopic data for [2-^{13}C]-cyclobutenone	S12
**Table S5**.	Experimental and computed spectroscopic constants for [3-^{13}C]-cyclobutenone	S13
**Figure S4.**	Data distribution plot for the least-squares fit of millimeter-wave spectroscopic data for [3-^{13}C]-cyclobutenone	S14
**Table S6**.	Experimental and computed spectroscopic constants for [4-^{13}C]-cyclobutenone	S15
**Figure S5.**	Data distribution plot for the least-squares fit of millimeter-wave spectroscopic data for [4-^{13}C]-cyclobutenone	S16
**Table S7.**	Experimental and computed spectroscopic constants for [^{18}O]-cyclobutenone	S17
**Figure S6.**	Data distribution plot for the least-squares fit of millimeter-wave spectroscopic data for [^{18}O]-cyclobutenone	S18
**Figure S7.**	Data distribution plot for the least-squares fit of millimeter-wave spectroscopic data for normal cyclobutenone, ν_{21}.	S19
**Figure S8.**	Data distribution plot for the least-squares fit of millimeter-wave spectroscopic data for normal cyclobutenone, $2\nu_{21}$.	S20
**Figure S9.**	Data distribution plot for the least-squares fit of millimeter-wave spectroscopic data for normal cyclobutenone, ν_{14}.	S21

Table of Contents		Page
**Figure S10.**	Data distribution plot for the least-squares fit of millimeter-wave spectroscopic data for normal cyclobutenone, ν_{20}.	S22
**Table S8.**	Determinable rotational constants calculated from A and S reductions, averaged determinable constants, and differences between determinable constants calculated from A and S reductions	S23
**Table S9.**	Differences between experimental determinable constants and those predicted by *xrefit*	S23
**Synthetic Details**		S24
**Experimental ^{1}H-NMR Spectrum**		S25
**References**		S26

**Additional Files Provided (zipped file)**	
CCSD(T) / cc-pCVTZ geometry optimization (*cyclobutenone_opt_ccsdt_cc-pCVTZ.out*)	Separate file
CCSD(T) / cc-pCVTZ VPT2 anharmonic frequency calculation (*cyclobutenone_ccsdt_cc-pCVTZ-freq_anharm.out*)	Separate file
CCSD(T) / cc-pCVTZ Isomass files containing computed distortion constants for isotopologues	Separate files
CCSD(T) / cc-pCVTZ magnetic calculations (*pyridine_magnetic_Iso##.out*)	Separate files
Xrefit r_e^{SE} structure output (*cyclobutenone_reSE.out*)	Separate file
Xrefit output without computational corrections (provides $\Delta_{i\,0}$) (*cyclobutenone_r0.out*)	Separate file
Xrefit output without electron-mass distribution correction (provides $\Delta_{i\,e}$ using vibration-rotation interaction correction only) (*cyclobutenone_reSE_sans_e.out*)	Separate file
Additional CFOUR optimization output files used to calculate BTE	Separate files
CCSD(T) / cc-pCVTZ Coriolis zeta values	Separate files
CCSD(T) / cc-pCVTZ cubic force constants	Separate file

Calculating the Determinable Constants

The ground-state rotational constants for both the S- and A-reduced Hamiltonians were converted to the "determinable constants" (A_0'', B_0'', C_0'') to account for the influence of centrifugal distortion using eq. (S1) – (S6).[1] The constants from each Hamiltonian reduction were then averaged to obtain the final determinable constants.

$$A_0'' = A_0^{(A)} + 2\Delta_J \quad \text{(S1)}$$

$$B_0'' = B_0^{(A)} + 2\Delta_J + \Delta_{JK} - 2\delta_J - 2\delta_K \quad \text{(S2)}$$

$$C_0'' = C_0^{(A)} + 2\Delta_J + \Delta_{JK} + 2\delta_J + 2\delta_K \quad \text{(S3)}$$

$$A_0'' = A_0^{(S)} + 2D_J + 6d_2 \quad \text{(S4)}$$

$$B_0'' = B_0^{(S)} + 2D_J + D_{JK} + 2d_1 + 4d_2 \quad \text{(S5)}$$

$$C_0'' = C_0^{(S)} + 2D_J + D_{JK} - 2d_1 + 4d_2 \quad \text{(S6)}$$

Ideally, the B_0'' values as determined from $B_0^{(A)}$ and $B_0^{(S)}$ are equal. The magnitude of the difference, if any, informs us as to the quality of the determinable constants. The differences between the B_0'' values for most isotopologues are well within two times the statistical uncertainties of the respective $B_0^{(A)}$ and $B_0^{(S)}$ values, and all of them are comparable in magnitude. This agreement provides confidence that both models employed in the least-squares fits are adequately treating the datasets and that the values obtained are suitable for use in the r_e^{SE} structure determination. See Table S8, below.

Calculating the Equilibrium Rotational Constant

$$B_e^{\beta} = B_0''^{\beta} + \frac{1}{2}\sum_i \alpha_i^{\beta} - \frac{m_e}{M_P} g^{\beta\beta} B^{\beta} \quad \text{(S7)}$$

where b refers to the rotational axis, B_0'' is the determinable rotational constant,[1] B_e is the equilibrium constant, the half-sum is the vibration-rotation interaction correction, $\frac{m_e}{M_p}$ is the electron-to-proton mass ratio, and g^{bb} is the corresponding magnetic g-tensor component.

Calculating the Best Theoretical Estimate (BTE)

A "best theoretical estimate" (BTE) for the structure of cyclobutenone uses the CCSD(T)/cc-pCV5Z structure as a basis, with four corrections that are evaluated using conventional methodology:[2-3]

1. Residual basis set effects beyond cc-pCV5Z: To estimate the correction needed to approach the infinite basis set limit, equilibrium structural parameters obtained with the cc-pCVXZ (X = T, Q, and 5) basis sets were extrapolated using the empirical exponential expression in Eq. (S8).[2-4]

$$R(x) = R(\infty) + Ae^{-Bx} \tag{S8}$$

R(x) are the values of the parameters obtained using the various basis sets (x = 3, 4, and 5), and R(∞) is the desired basis set limit estimate. Using three basis sets (x = 3, 4, and 5), the system of equations using Eq. (S8) can be solved, yielding Eq. (S9).[2]

$$R(\infty) = -\frac{R(4)^2 - R(3)R(5)}{R(3) + R(5) - 2R(4)} \tag{S9}$$

The correction to the structure due to a finite basis set is estimated by Eq. (S10).

$$\Delta R(\text{basis}) = R(\infty) - R(\text{CCSD(T)/cc-pCV5Z}) \tag{S10}$$

2. Residual electron correlation effects beyond the CCSD(T) treatment: Residual correlation effects are assessed by doing geometry optimizations at the CCSDT(Q) level[5] and then estimating the correlation correction in Eq. (S11).

$$\Delta R(\text{cor}) = R(\text{CCSDT(Q)}) - R(\text{CCSD(T)}) \tag{S11}$$

   As calculations at the CCSDT(Q) level of theory are computationally demanding, these two calculations are obtained with the cc-pVDZ basis, in the frozen-core approximation.

3. Effects of scalar (mass-velocity and Darwin) relativistic effects: The relativistic corrections are obtained by subtraction of the equilibrium parameters obtained with a standard non-relativistic calculation from those obtained with the X2C-1e variant of coupled-cluster theory,[6-8] as shown in Eq. (S12).

$$\Delta \text{R(rel)} = R(\text{CCSD(T)/cc-pCVTZ})_{\text{SFX2C-1e}} - R(\text{CCSD(T)/cc-pCVTZ})_{NR} \tag{S12}$$

The relativistic calculation was performed with the re-contracted basis set XC2-pCVTZ, the non-relativistic calculation was performed with the cc-pVTZ basis set. All electrons were correlated in both calculations. (Keyword: RELATIVISTIC=X2C1E)

4. The diagonal Born-Oppenheimer correction (DBOC): The diagonal Born-Oppenheimer correction (DBOC)[9-10] is obtained from Eq. (S13).

$$\Delta R(\text{DBOC}) = R(\text{SCF/cc-pVTZ})_{DBOC} - R(\text{SCF/cc-pVTZ})_{NR} \tag{S13}$$

   Here, the first value is obtained by minimizing the DBOC-corrected SCF energy with respect to nuclear positions, and the latter is again the traditional calculation. (Keyword: DBOC=ON)

   More details are available concerning corrections for non-Born-Oppenheimer behavior.[2, 11-12]

The sum of the above-described corrections is used to obtain the best equilibrium structural parameters, given by Eq. (S14).

$$\Delta R(\text{best}) = \Delta R(\text{basis}) + \Delta R(\text{cor}) + \Delta R(\text{rel}) + \Delta R(\text{DBOC}) \quad \text{(S14)}$$

The sum of the corrections, ΔR(total), is applied to the CCSD(T)/cc-pCV5Z structural parameters.

Table S1. Corrections (Δ*R*) Applied to Computed Structural Parameters (CCSD(T)/cc-pCV5Z) to Afford the Best Theoretical Estimate (BTE) for Cyclobutenone [a]

Parameter	Δ*R*(basis)	Δ*R*(rel)	Δ*R*(corr)	Δ*R*(DBOC)	Δ*R*(total)
$R_{O–C1}$ (Å)	−0.00001	−0.00015	0.00106	0.00005	0.00095
$R_{C1–C2}$ (Å)	−0.00039	−0.00014	0.00031	0.00004	−0.00018
$R_{C1–C4}$ (Å)	−0.00043	−0.00010	0.00101	0.00001	0.00049
$R_{C2–C3}$ (Å)	−0.00019	−0.00025	0.00101	0.00004	0.00061
$R_{C2–H}$ (Å)	−0.00008	−0.00010	0.00015	0.00015	0.00012
$R_{C3–H}$ (Å)	−0.00007	−0.00011	0.00009	0.00016	0.00008
$R_{C4–H}$ (Å)	−0.00012	−0.00010	0.00021	0.00013	0.00013
$\theta_{O–C1–C2}$ (°)	−0.00586	0.00205	−0.01417	−0.00185	−0.01983
$\theta_{O–C1–C4}$ (°)	−0.06322	0.00330	0.01971	−0.00172	−0.04194
$\theta_{C1–C2–C3}$ (°)	−0.00909	0.00273	0.01451	−0.00250	0.00565
$\theta_{H–C2–C1}$ (°)	−0.05603	−0.00047	−0.00957	0.00141	−0.06466
$\theta_{H–C4–C1}$ (°)	−0.01330	0.00027	0.00182	−0.00281	−0.01402
$\theta_{H–C3–C2}$ (°)	−0.00953	−0.00246	0.01111	−0.00215	−0.00303
$\lvert\theta_{H–C4–C1–O}\rvert$ (°)	0.00020	0.00192	0.00173	0.00111	0.00111

[a] Exact values were used for all mathematical functions, but values in table are rounded to one decimal more than provided in structural determination for legibility, resulting in apparent discrepancies between sum of rounded corrections shown and rounded Δ*R*(total).

Distance (*R*) in Ångstrom (Å); angle (θ) in degree (°).

Isotopologue Numbering

normal cyclobutenone: 01
[1-^{13}C]-cyclobutenone: 02
[2-^{13}C]-cyclobutenone: 03
[3-^{13}C]-cyclobutenone: 04
[4-^{13}C]-cyclobutenone: 05
[^{18}O]-cyclobutenone: 06

Overview of tables of spectroscopic constants and figures of data distribution plots. For archival purposes, data for all isotopologues are included in the Supporting Information, even if small portions of these data have been included in the manuscript.

Table S2. Experimental and computed spectroscopic constants for the normal isotopologue of cyclobutenone

	S Reduction, I^r representation			A Reduction, I^r representation	
	Experimental [a]	CCSD(T) [b]		Experimental [a]	CCSD(T) [b]
A_0 (MHz)	12548.26963 (16)	12482.6	A_0 (MHz)	12548.26856 (16)	12482.6
B_0 (MHz)	5307.559753 (31)	5276.0	B_0 (MHz)	5307.567214 (32)	5276.0
C_0 (MHz)	3820.176505 (28)	3797.7	C_0 (MHz)	3820.169613 (29)	3797.7
D_J (kHz)	0.5792371 (80)	0.562976	Δ_J (kHz)	0.7464252 (89)	0.72317800
D_{JK} (kHz)	6.477489 (37)	6.319030	Δ_{JK} (kHz)	5.474062 (37)	5.35782
D_K (kHz)	1.48957 (82)	1.5197400	Δ_K (kHz)	2.32524 (84)	2.3207500
d_1 (kHz)	−0.1994048 (32)	−0.1937720	δ_J (kHz)	0.1994074 (32)	0.1937720
d_2 (kHz)	−0.0836013 (12)	−0.0801010	δ_K (kHz)	3.590471 (51)	3.4734700
H_J (Hz)	0.00005694 (75)	0.0000241	Φ_J (Hz)	0.0001821 (10)	0.0001522
H_{JK} (Hz)	−0.001170 (13)	−0.0004468	Φ_{JK} (Hz)	0.005181 (16)	0.0055138
H_{KJ} (Hz)	0.009740 (40)	0.0077109	Φ_{KJ} (Hz)	−0.013509 (57)	−0.0140802
H_K (Hz)	0.0033 (12)	0.0023157	Φ_K (Hz)	0.0195 (12)	0.0180181
h_1 (Hz)	0.00004749 (44)	0.0000405	ϕ_J (Hz)	0.00008970 (46)	0.0000793
h_2 (Hz)	0.00006287 (28)	0.0000641	ϕ_{JK} (Hz)	0.003082 (12)	0.0031682
h_3 (Hz)	0.000042010 (83)	0.0000388	ϕ_K (Hz)	0.03640 (10)	0.0342915
N_{lines} [c]	3848		N_{lines} [c]	3848	
σ_{fit} (MHz)	0.031		σ_{fit} (MHz)	0.031	
κ [d]	−0.659173	−0.659570	κ [d]	−0.659170	−0.659570
Δ_i [d]	−3.201452 (1)	−3.199945	Δ_i [d]	−3.201083 (1)	−3.199945

[a] Includes transitions from previous work.[13]
[b] Evaluated using the cc-pCVTZ basis set.
[c] Number of independent transitions.
[d] Inertial defect (Δ_i) and κ calculated from B_0 constants using PLANM.

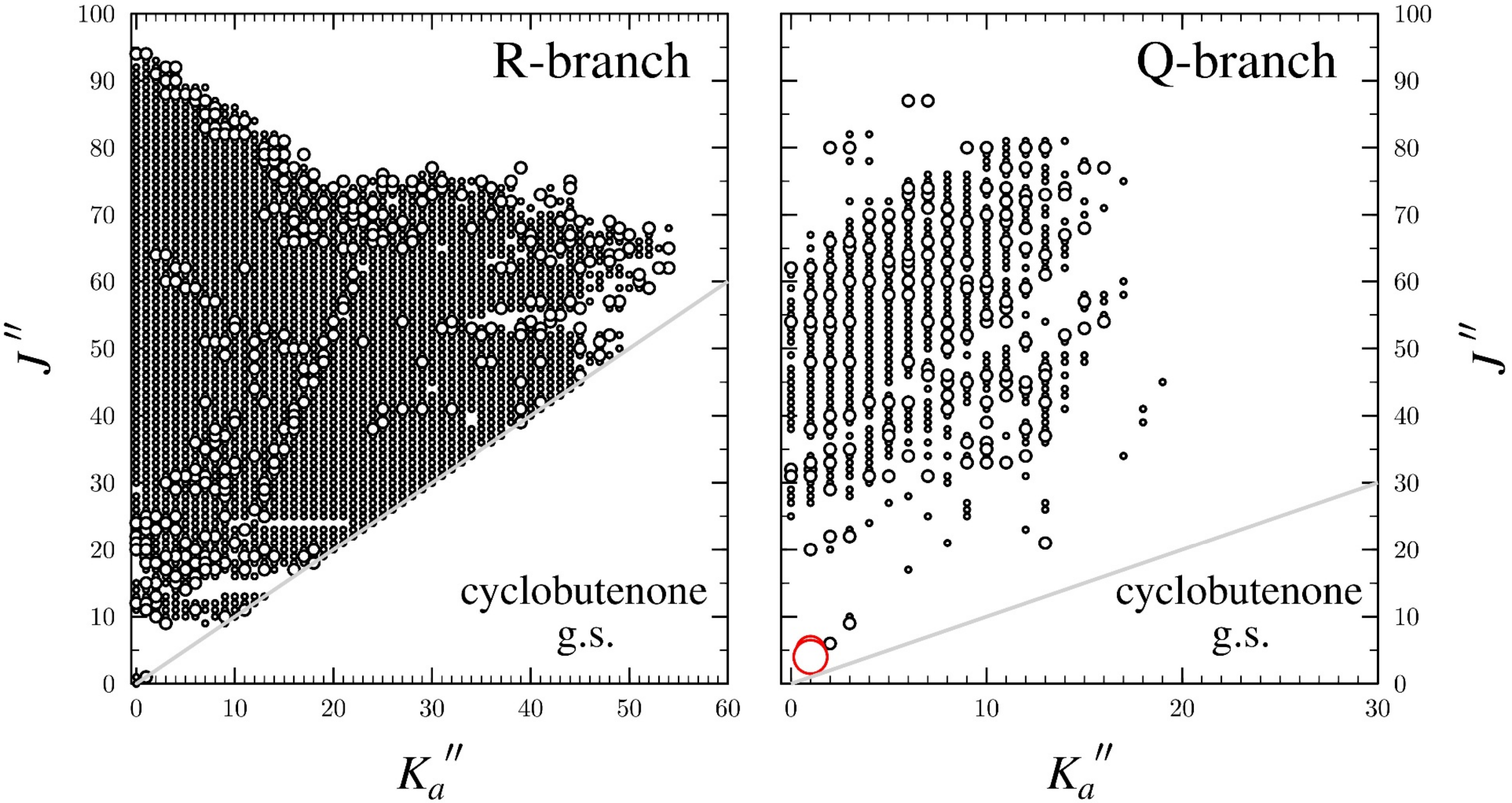

Figure S1. Data distribution plot for the least-squares fit of spectroscopic data for the vibrational ground state of cyclobutenone. The size of the outlined circle is proportional to the value of $|(f_{\text{obs.}} - f_{\text{calc.}})/\delta f|$, where δf is the frequency measurement uncertainty, and two of the quotient values are equal to or greater than 3× their nominal measurement uncertainty and are shown in red. The data set includes all of the microwave transitions from Carrillo *et al.*,[13] two of which are the red transitions.

Table S3. Experimental and computed spectroscopic constants for [1-^{13}C]-cyclobutenone [a]

	S Reduction, I^r representation			A Reduction, I^r representation	
	Experimental	CCSD(T) [b]		Experimental	CCSD(T) [b]
A_0 (MHz)	12548.7694 (26)	12483.1	A_0 (MHz)	12548.7663 (26)	12483.1
B_0 (MHz)	5290.94658 (16)	5259.4	B_0 (MHz)	5290.95388 (16)	5259.4
C_0 (MHz)	3811.62791 (11)	3789.2	C_0 (MHz)	3811.62114 (10)	3789.2
D_J (kHz)	0.582110 (27)	0.565980	Δ_J (kHz)	0.746825 (29)	0.72381100
D_{JK} (kHz)	6.39195 (15)	6.236390	Δ_{JK} (kHz)	5.40342 (14)	5.28940
D_K (kHz)	1.5542 (95)	1.5975500	Δ_K (kHz)	2.3685 (95)	2.3867100
d_1 (kHz)	−0.199344 (15)	−0.1937160	δ_J (kHz)	0.199334 (14)	0.1937160
d_2 (kHz)	−0.0823662 (75)	−0.0789156	δ_K (kHz)	3.56242 (32)	3.4463900
H_J (Hz)	[0.0000274]	0.0000274	Φ_J (Hz)	[0.0001522]	0.0001522
H_{JK} (Hz)	[−0.0005451]	−0.0005451	Φ_{JK} (Hz)	[0.0053117]	0.0053117
H_{KJ} (Hz)	[0.0074618]	0.0074618	Φ_{KJ} (Hz)	[−0.0139341]	−0.0139341
H_K (Hz)	[0.0026464]	0.0026464	Φ_K (Hz)	[0.0180605]	0.0180605
h_1 (Hz)	[0.0000416]	0.0000416	ϕ_J (Hz)	[0.0000794]	0.0000794
h_2 (Hz)	[0.0000624]	0.0000624	ϕ_{JK} (Hz)	[0.0030729]	0.0030729
h_3 (Hz)	[0.0000378]	0.0000378	ϕ_K (Hz)	[0.0339416]	0.0339416
N_{lines} [c]	649		N_{lines} [c]	649	
σ_{fit} (MHz)	0.043		σ_{fit} (MHz)	0.043	
κ [d]	−0.661372	−0.661786	κ [d]	−0.661369	−0.661786
Δ_i [d]	−3.202128 (10)	−3.202139	Δ_i [d]	−3.201770 (9)	−3.202139

[a] Values in brackets held constant at the CCSD(T)/cc-pCVTZ value.
[b] Evaluated using the cc-pCVTZ basis set.
[c] Number of independent transitions.
[d] Inertial defect (Δ_i) and κ calculated from B_0 constants using PLANM.

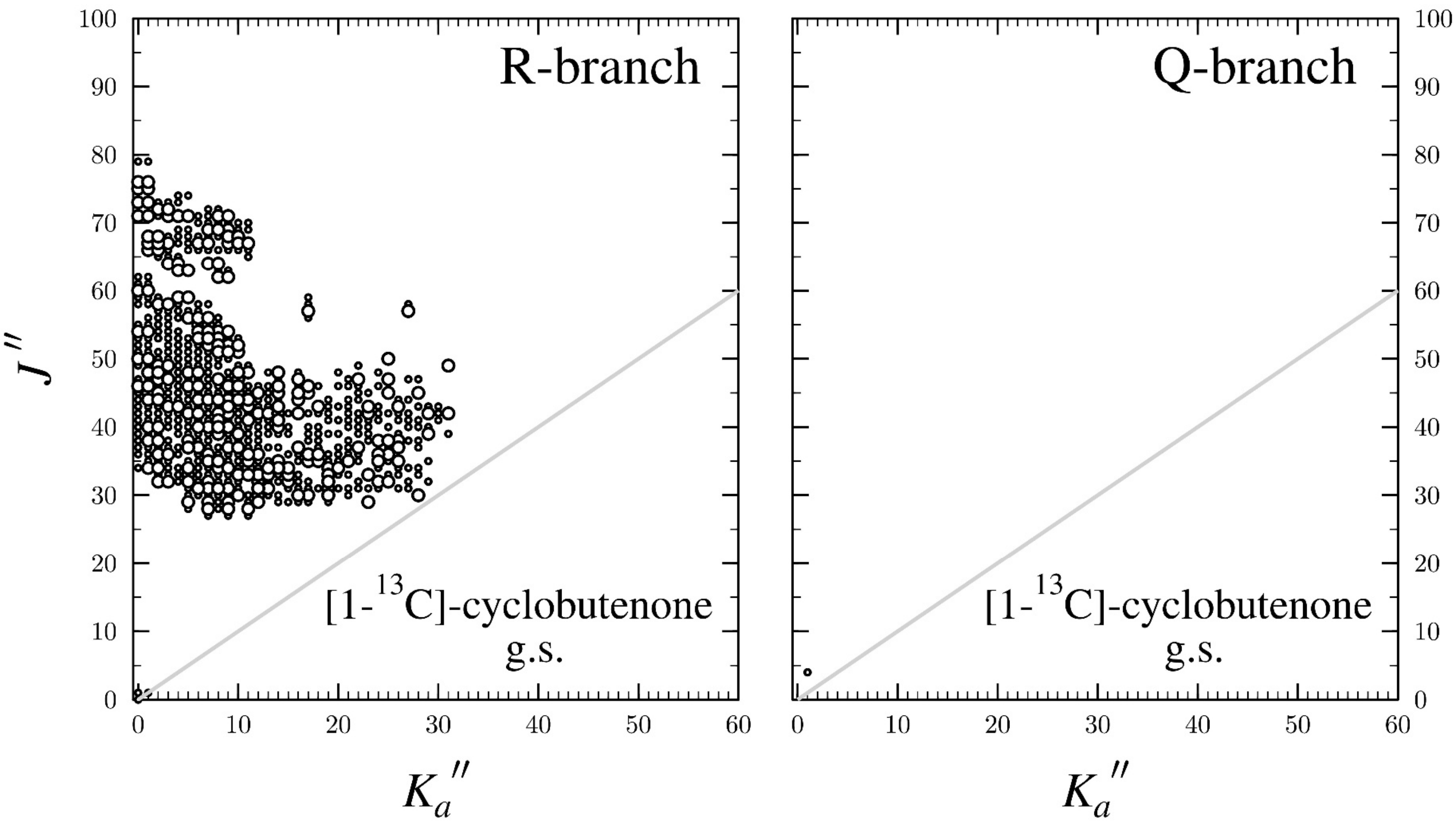

Figure S2. Data distribution plot for the least-squares fit of millimeter-wave spectroscopic data for [1-^{13}C]-cyclobutenone, ground vibrational state. Black circles are measurements from the current work. The size of the outlined circle is proportional to the value of $|(f_{obs.} - f_{calc.})/\delta f|$, where δf is the frequency measurement uncertainty (50 kHz), and no quotient values are larger than three.

Table S4. Experimental and computed spectroscopic constants for [2-^{13}C]-cyclobutenone [a]

	S Reduction, I^r representation			A Reduction, I^r representation	
	Experimental	CCSD(T) [b]		Experimental	CCSD(T) [b]
A_0 (MHz)	12219.0636 (20)	12154.9	A_0 (MHz)	12219.0606 (20)	12154.9
B_0 (MHz)	5291.74197 (15)	5260.2	B_0 (MHz)	5291.74906 (15)	5260.2
C_0 (MHz)	3780.98845 (10)	3758.7	C_0 (MHz)	3780.98186 (10)	3758.7
D_J (kHz)	0.566293 (18)	0.550686	Δ_J (kHz)	0.738600 (23)	0.71591000
D_{JK} (kHz)	6.36740 (12)	6.209810	Δ_{JK} (kHz)	5.33322 (13)	5.21846
D_K (kHz)	1.2080 (46)	1.2318900	Δ_K (kHz)	2.0634 (46)	2.0580100
d_1 (kHz)	−0.200466 (12)	−0.1947970	δ_J (kHz)	0.200444 (12)	0.1947970
d_2 (kHz)	−0.0861700 (70)	−0.0826123	δ_K (kHz)	3.50568 (28)	3.3934200
H_J (Hz)	[0.0000203]	0.0000203	Φ_J (Hz)	[0.0001515]	0.0001515
H_{JK} (Hz)	[−0.0004164]	−0.0004164	Φ_{JK} (Hz)	[0.0054355]	0.0054355
H_{KJ} (Hz)	[0.0076308]	0.0076308	Φ_{KJ} (Hz)	[−0.0138446]	−0.0138446
H_K (Hz)	[0.0018464]	0.0018464	Φ_K (Hz)	[0.0173386]	0.0173386
h_1 (Hz)	[0.0000387]	0.0000387	ϕ_J (Hz)	[0.0000791]	0.0000791
h_2 (Hz)	[0.0000656]	0.0000656	ϕ_{JK} (Hz)	[0.0031169]	0.0031169
h_3 (Hz)	[0.0000404]	0.0000404	ϕ_K (Hz)	[0.0318871]	0.0318871
N_{lines} [c]	646		N_{lines} [c]	646	
σ_{fit} (MHz)	0.043		σ_{fit} (MHz)	0.043	
κ [d]	−0.641920	−0.642338	κ [d]	−0.641917	−0.642338
Δ_i [d]	−3.200018 (8)	−3.198420	Δ_i [d]	−3.199667 (8)	−3.198420

[a] Values in brackets held constant at the CCSD(T)/cc-pCVTZ value.
[b] Evaluated using the cc-pCVTZ basis set.
[c] Number of independent transitions.
[d] Inertial defect (Δ_i) and κ calculated from B_0 constants using PLANM.

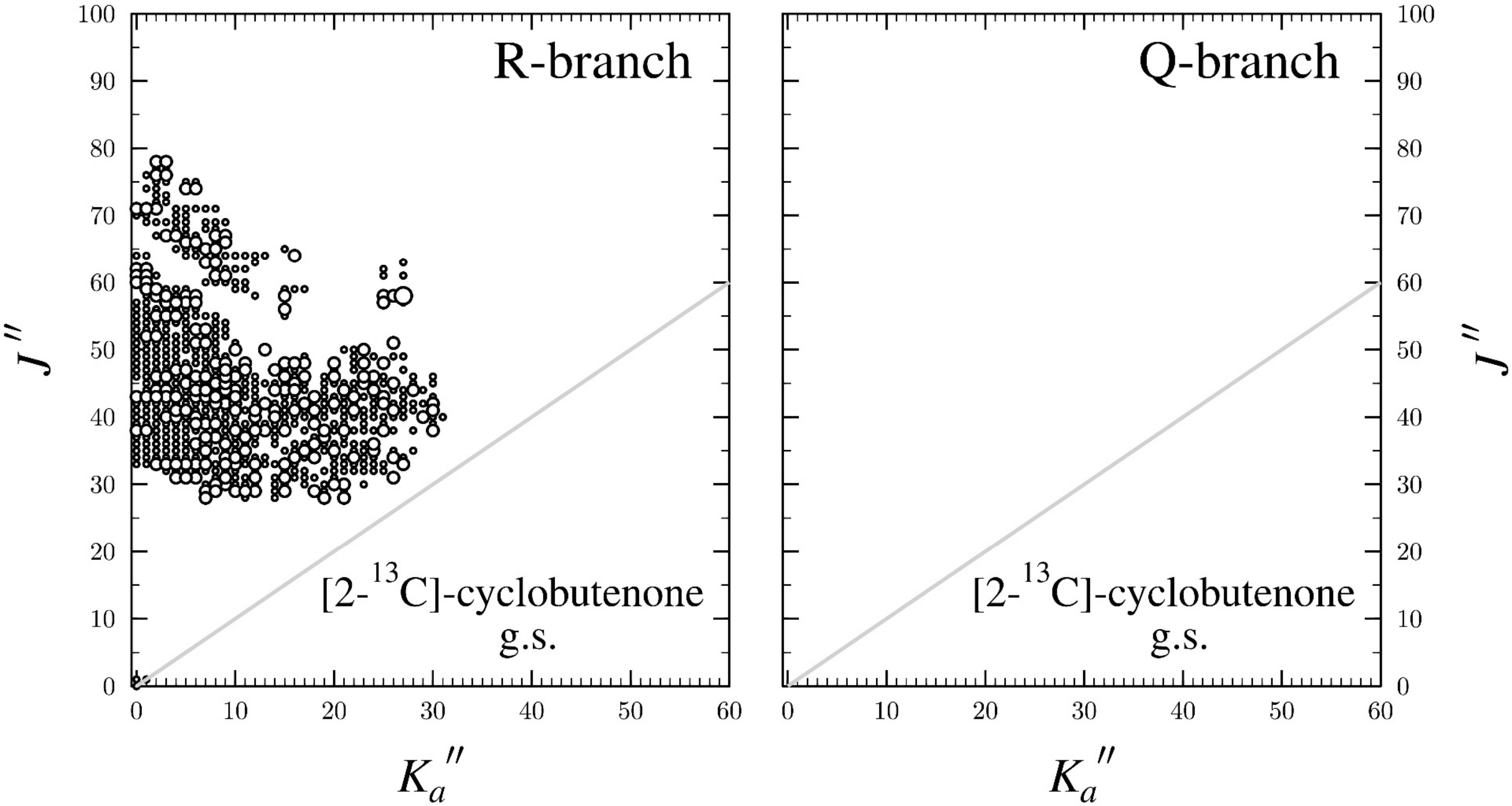

Figure S3. Data distribution plot for the least-squares fit of millimeter-wave spectroscopic data for [2-^{13}C]-cyclobutenone, ground vibrational state. Black circles are measurements from the current work. The size of the outlined circle is proportional to the value of $|(f_{obs.} - f_{calc.})/\delta f|$, where δf is the frequency measurement uncertainty, and no quotient values are larger than three.

Table S5. Experimental and computed spectroscopic constants for [3-^{13}C]-cyclobutenone [a]

	S Reduction, I^r representation			A Reduction, I^r representation	
	Experimental	CCSD(T) [b]		Experimental	CCSD(T) [b]
A_0 (MHz)	12546.3671 (24)	12480.6	A_0 (MHz)	12546.3641 (24)	12480.6
B_0 (MHz)	5189.18770 (20)	5158.4	B_0 (MHz)	5189.19468 (20)	5158.4
C_0 (MHz)	3758.31706 (12)	3736.3	C_0 (MHz)	3758.31052 (12)	3736.3
D_J (kHz)	0.560073 (26)	0.544307	Δ_J (kHz)	0.712847 (34)	0.69062500
D_{JK} (kHz)	6.13345 (13)	5.981560	Δ_{JK} (kHz)	5.21651 (15)	5.10365
D_K (kHz)	1.8712 (61)	1.8872900	Δ_K (kHz)	2.6283 (61)	2.6188800
d_1 (kHz)	−0.188466 (18)	−0.1831290	δ_J (kHz)	0.188448 (17)	0.1831290
d_2 (kHz)	−0.0764030 (78)	−0.0731591	δ_K (kHz)	3.44849 (35)	3.3338600
H_J (Hz)	[0.0000281]	0.0000281	Φ_J (Hz)	[0.0001458]	0.0001458
H_{JK} (Hz)	[−0.0003236]	−0.0003236	Φ_{JK} (Hz)	[0.005129]	0.0051290
H_{KJ} (Hz)	[0.0061321]	0.0061321	Φ_{KJ} (Hz)	[−0.0138095]	−0.0138095
H_K (Hz)	[0.003777]	0.0037770	Φ_K (Hz)	[0.0181484]	0.0181484
h_1 (Hz)	[0.0000412]	0.0000412	ϕ_J (Hz)	[0.000075]	0.0000750
h_2 (Hz)	[0.0000589]	0.0000589	ϕ_{JK} (Hz)	[0.0029682]	0.0029682
h_3 (Hz)	[0.0000338]	0.0000338	ϕ_K (Hz)	[0.0332776]	0.0332776
N_{lines} [c]	628		N_{lines} [c]	628	
σ_{fit} (MHz)	0.045		σ_{fit} (MHz)	0.045	
κ [d]	−0.674360	−0.674737	κ [d]	−0.674357	−0.674737
Δ_i [d]	−3.202180 (10)	−3.203321	Δ_i [d]	−3.201825 (10)	−3.203321

[a] Values in brackets held constant at the CCSD(T)/cc-pCVTZ value.
[b] Evaluated using the cc-pCVTZ basis set.
[c] Number of independent transitions.
[d] Inertial defect (Δ_i) and κ calculated from B_0 constants using PLANM.

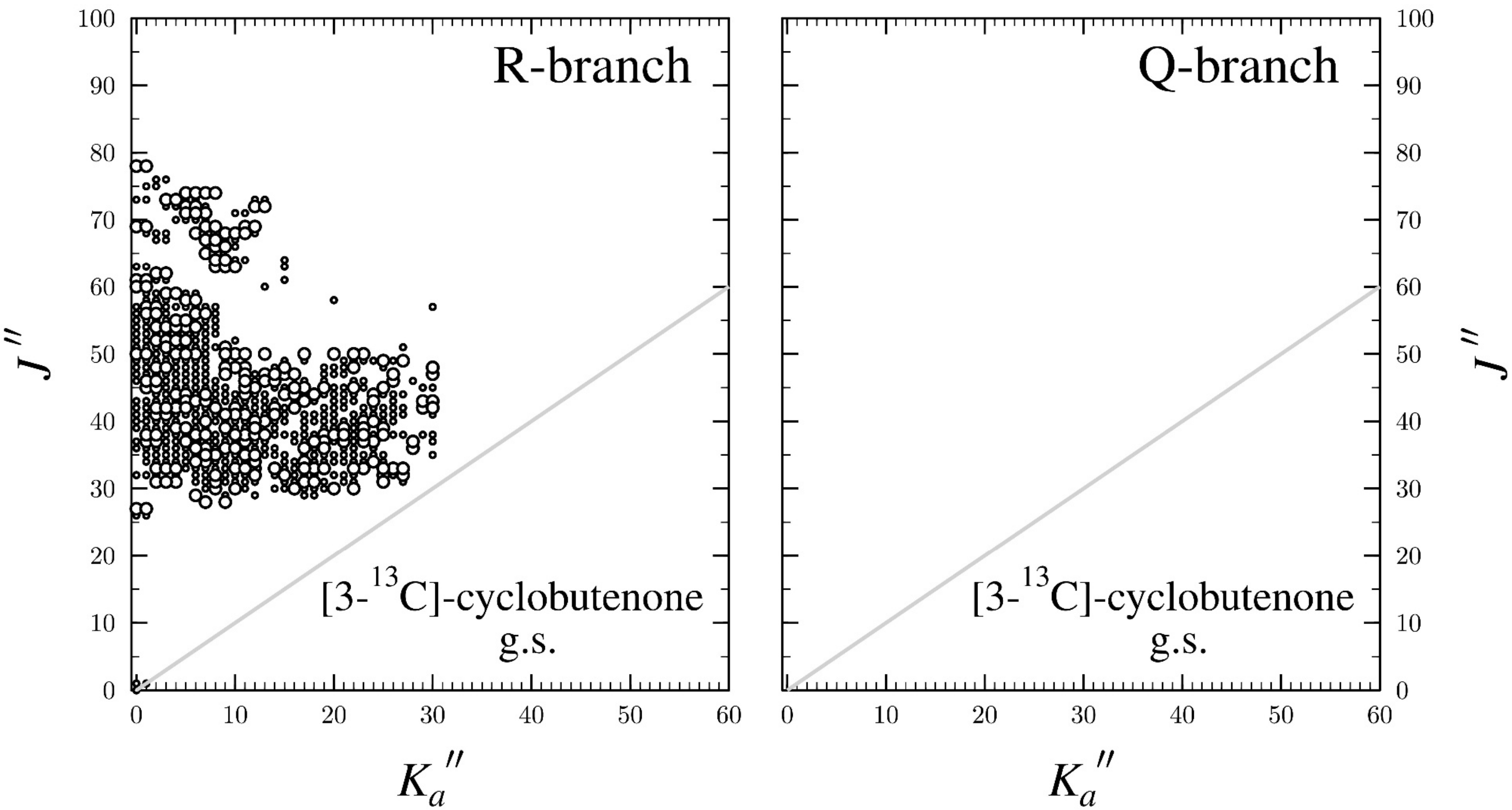

Figure S4. Data distribution plot for the least-squares fit of millimeter-wave spectroscopic data for [3-^{13}C]-cyclobutenone, ground vibrational state. Black circles are measurements from the current work. The size of the outlined circle is proportional to the value of $|(f_{obs.} - f_{calc.})/\delta f|$, where δf is the frequency measurement uncertainty (50 kHz), and no quotient values are larger than three.

Table S6. Experimental and computed spectroscopic constants for [4-^{13}C]-cyclobutenone [a]

	S Reduction, I^r representation			A Reduction, I^r representation	
	Experimental	CCSD(T) [b]		Experimental	CCSD(T) [b]
A_0 (MHz)	12187.4361 (20)	12123.3	A_0 (MHz)	12187.4332 (20)	12123.3
B_0 (MHz)	5292.24148 (17)	5260.7	B_0 (MHz)	5292.24873 (17)	5260.7
C_0 (MHz)	3778.18700 (12)	3756.0	C_0 (MHz)	3778.18035 (12)	3756.0
D_J (kHz)	0.568478 (18)	0.552824	Δ_J (kHz)	0.742811 (23)	0.71994300
D_{JK} (kHz)	6.43032 (12)	6.271760	Δ_{JK} (kHz)	5.38408 (12)	5.26905
D_K (kHz)	1.1833 (42)	1.1992600	Δ_K (kHz)	2.0480 (43)	2.0348500
d_1 (kHz)	−0.201221 (12)	−0.1955130	δ_J (kHz)	0.201211 (12)	0.1955130
d_2 (kHz)	−0.0871737 (75)	−0.0835592	δ_K (kHz)	3.52482 (30)	3.4109200
H_J (Hz)	[0.0000209]	0.0000209	Φ_J (Hz)	[0.0001485]	0.0001485
H_{JK} (Hz)	[−0.0006697]	−0.0006697	Φ_{JK} (Hz)	[0.0053096]	0.0053096
H_{KJ} (Hz)	[0.0084101]	0.0084101	Φ_{KJ} (Hz)	[−0.0134345]	−0.0134345
H_K (Hz)	[0.0011615]	0.0011615	Φ_K (Hz)	[0.0168992]	0.0168992
h_1 (Hz)	[0.0000369]	0.0000369	ϕ_J (Hz)	[0.0000782]	0.0000782
h_2 (Hz)	[0.0000638]	0.0000638	ϕ_{JK} (Hz)	[0.0030533]	0.0030533
h_3 (Hz)	[0.0000413]	0.0000413	ϕ_K (Hz)	[0.0321907]	0.0321907
N_{lines} [c]	663		N_{lines} [c]	663	
σ_{fit} (MHz)	0.045		σ_{fit} (MHz)	0.045	
κ [d]	−0.639907	−0.640338	κ [d]	−0.639904	−0.640338
Δ_i [d]	−3.199228 (9)	−3.201011	Δ_i [d]	−3.198871 (9)	−3.201011

[a] Values in brackets held constant at the CCSD(T)/cc-pCVTZ value.

[b] Evaluated using the cc-pCVTZ basis set.

[c] Number of independent transitions.

[d] Inertial defect (Δ_i) and κ calculated from B_0 constants using PLANM.

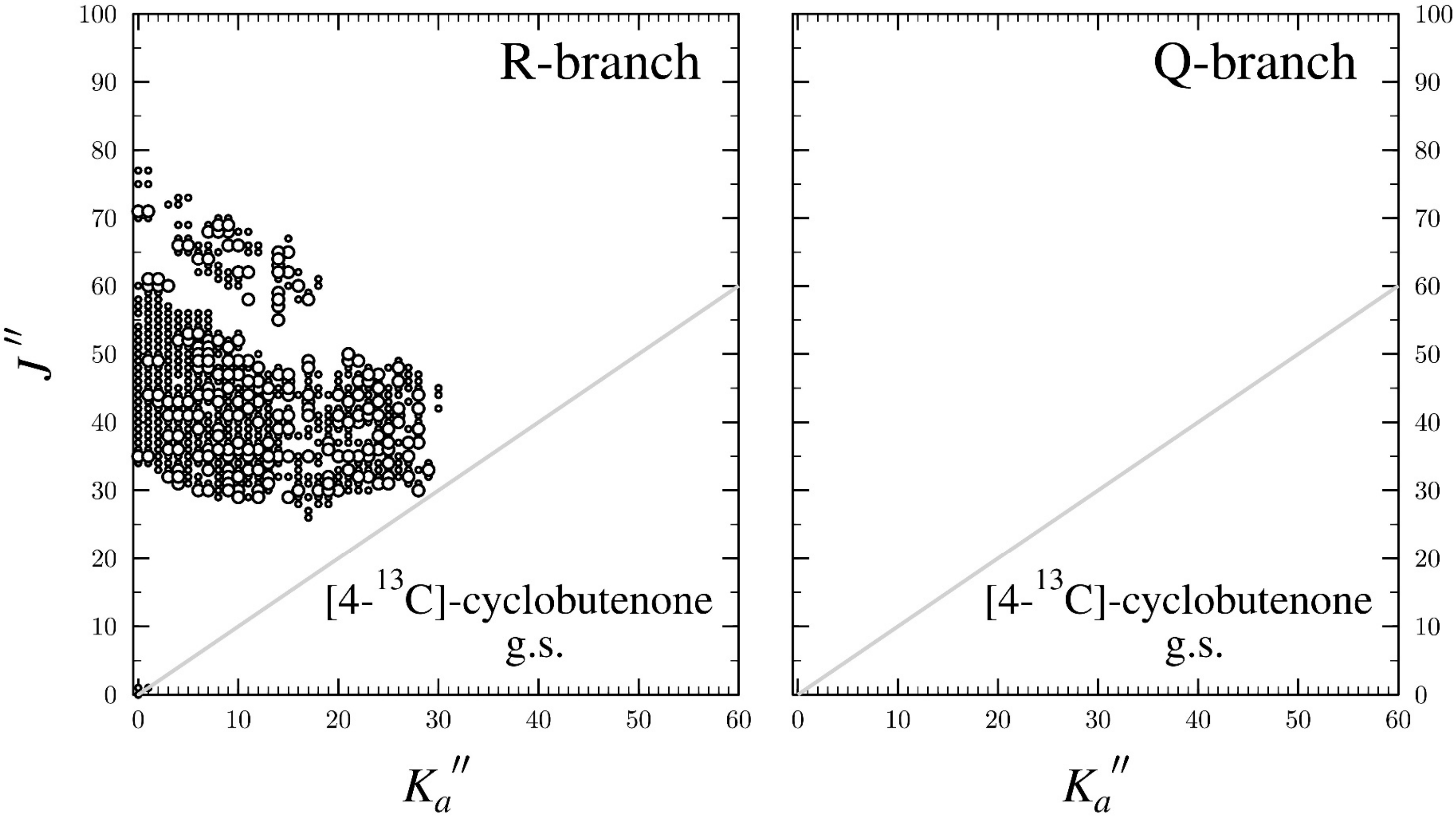

Figure S5. Data distribution plot for the least-squares fit of millimeter-wave spectroscopic data for [4-^{13}C]-cyclobutenone, ground vibrational state. Black circles are measurements from the current work. The size of the outlined circle is proportional to the value of $|(f_{obs.} - f_{calc.})/\delta f|$, where δf is the frequency measurement uncertainty (50 kHz), and no quotient values are larger than three.

Table S7. Experimental and computed spectroscopic constants for [^{18}O]-cyclobutenone [a]

	S Reduction, I^r representation			A Reduction, I^r representation	
	Experimental	CCSD(T) [b]		Experimental	CCSD(T) [b]
A_0 (MHz)	12547.805 (48)	12482.0	A_0 (MHz)	12547.792 (48)	12482.0
B_0 (MHz)	4993.4017 (14)	4963.7	B_0 (MHz)	4993.4087 (14)	4963.7
C_0 (MHz)	3654.59649 (48)	3633.1	C_0 (MHz)	3654.59013 (48)	3633.1
D_J (kHz)	0.5747 (43)	0.512870	Δ_J (kHz)	0.6974 (44)	0.64573800
D_{JK} (kHz)	5.176 (65)	5.906560	Δ_{JK} (kHz)	4.580 (66)	5.10936
D_K (kHz)	[1.97962]	1.9796200	Δ_K (kHz)	[2.64395]	2.6439500
d_1 (kHz)	−0.1925 (22)	−0.1670710	δ_J (kHz)	0.1875 (22)	0.1670710
d_2 (kHz)	[−0.0664335]	−0.0664335	δ_K (kHz)	[3.29676]	3.2967600
H_J (Hz)	[0.0000205]	0.0000205	Φ_J (Hz)	[0.0001249]	0.0001249
H_{JK} (Hz)	[−0.0002753]	−0.0002753	Φ_{JK} (Hz)	[0.0050539]	0.0050539
H_{KJ} (Hz)	[0.0067303]	0.0067303	Φ_{KJ} (Hz)	[−0.0126004]	−0.0126004
H_K (Hz)	[0.0031167]	0.0031167	Φ_K (Hz)	[0.0170138]	0.0170138
h_1 (Hz)	[0.0000348]	0.0000348	ϕ_J (Hz)	[0.0000648]	0.0000648
h_2 (Hz)	[0.0000522]	0.0000522	ϕ_{JK} (Hz)	[0.0028879]	0.0028879
h_3 (Hz)	[0.00003]	0.0000300	ϕ_K (Hz)	[0.0350176]	0.0350176
N_{lines} [c]	57		N_{lines} [c]	57	
σ_{fit} (MHz)	0.045		σ_{fit} (MHz)	0.045	
κ [d]	−0.698915	−0.699262	κ [d]	−0.698912	−0.699262
Δ_i [d]	−3.19979 (16)	−3.19953	Δ_i [d]	−3.19945 (16)	−3.19953

[a] Values in brackets held constant at the CCSD(T)/cc-pCVTZ value.
[b] Evaluated using the cc-pCVTZ basis set.
[c] Number of independent transitions.
[d] Inertial defect (Δ_i) and κ calculated from B_0 constants using PLANM.

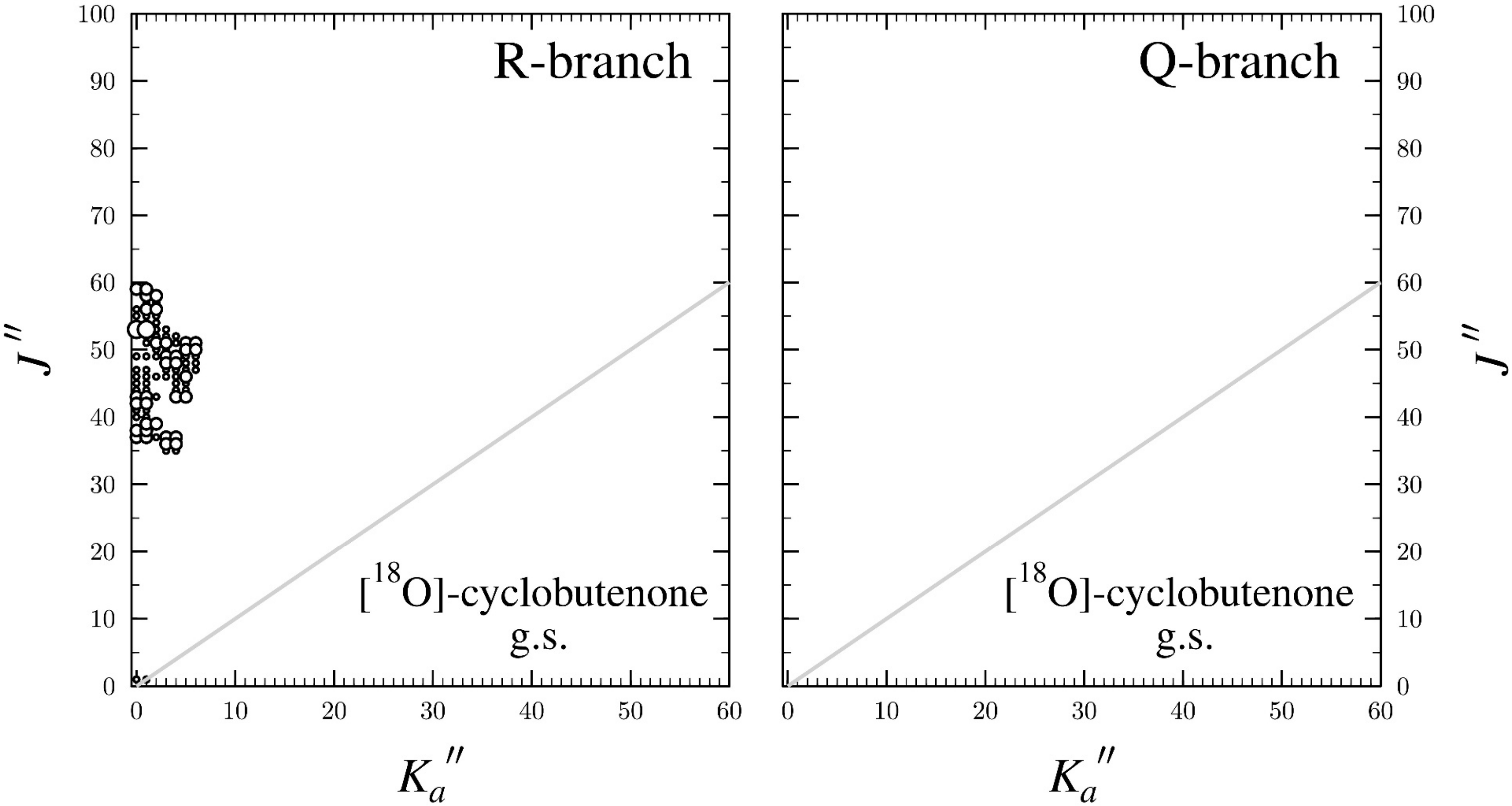

Figure S6. Data distribution plot for the least-squares fit of millimeter-wave spectroscopic data for [^{18}O]-cyclobutenone, ground vibrational state. Black (all) circles are measurements from the current work. The size of the outlined circle is proportional to the value of $|(f_{obs.} - f_{calc.})/\delta f|$, where δf is the frequency measurement uncertainty (50 kHz), and no quotient values are larger than three.

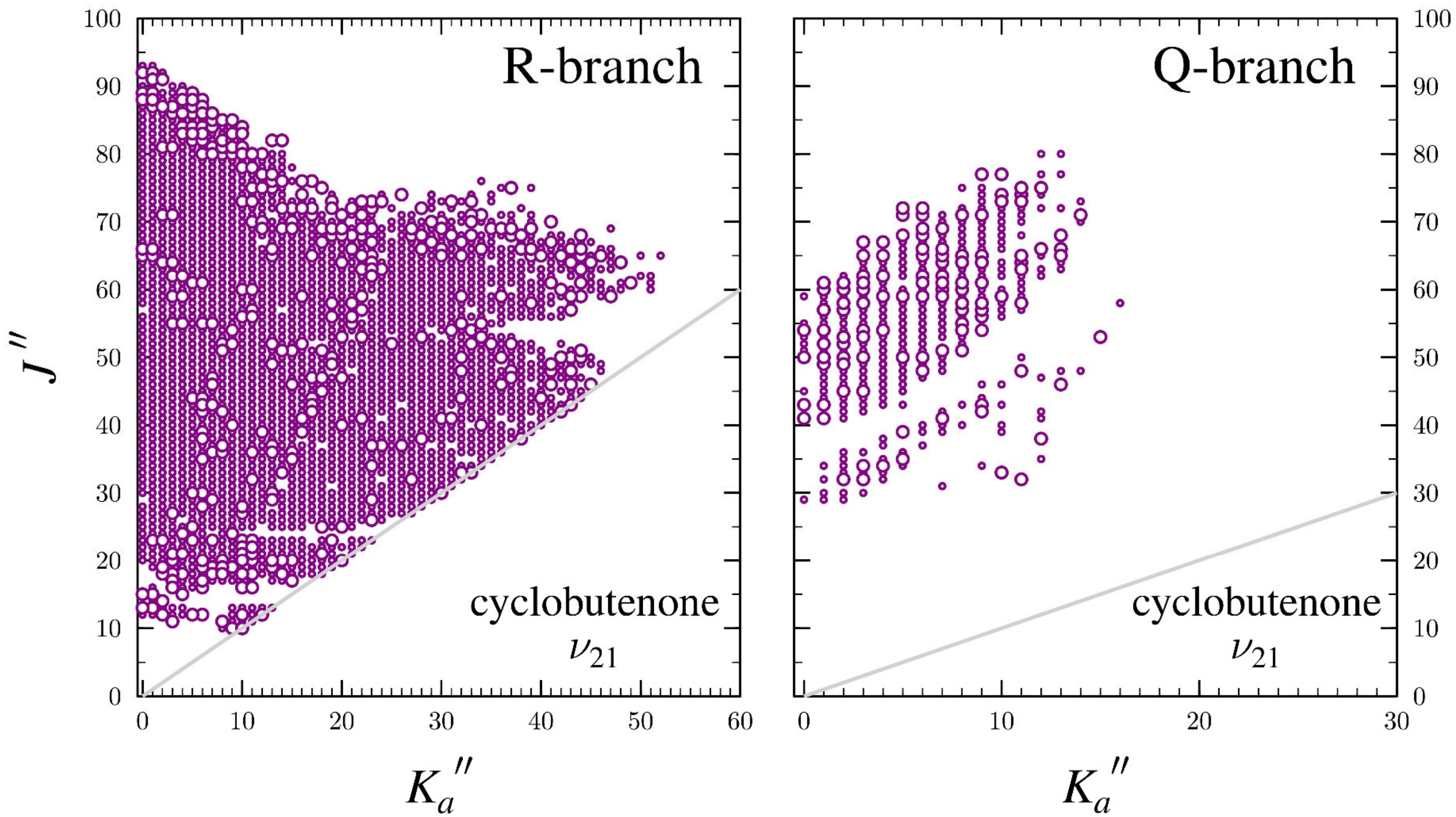

Figure S7. Data distribution plot for the least-squares fit of millimeter-wave spectroscopic data for cyclobutenone, ν_{21}. Purple circles (all) are measurements from the current work. The size of the outlined circle is proportional to the value of $|(f_{obs.} - f_{calc.})/\delta f|$, where δf is the frequency measurement uncertainty (50 kHz), and no quotient values are larger than three.

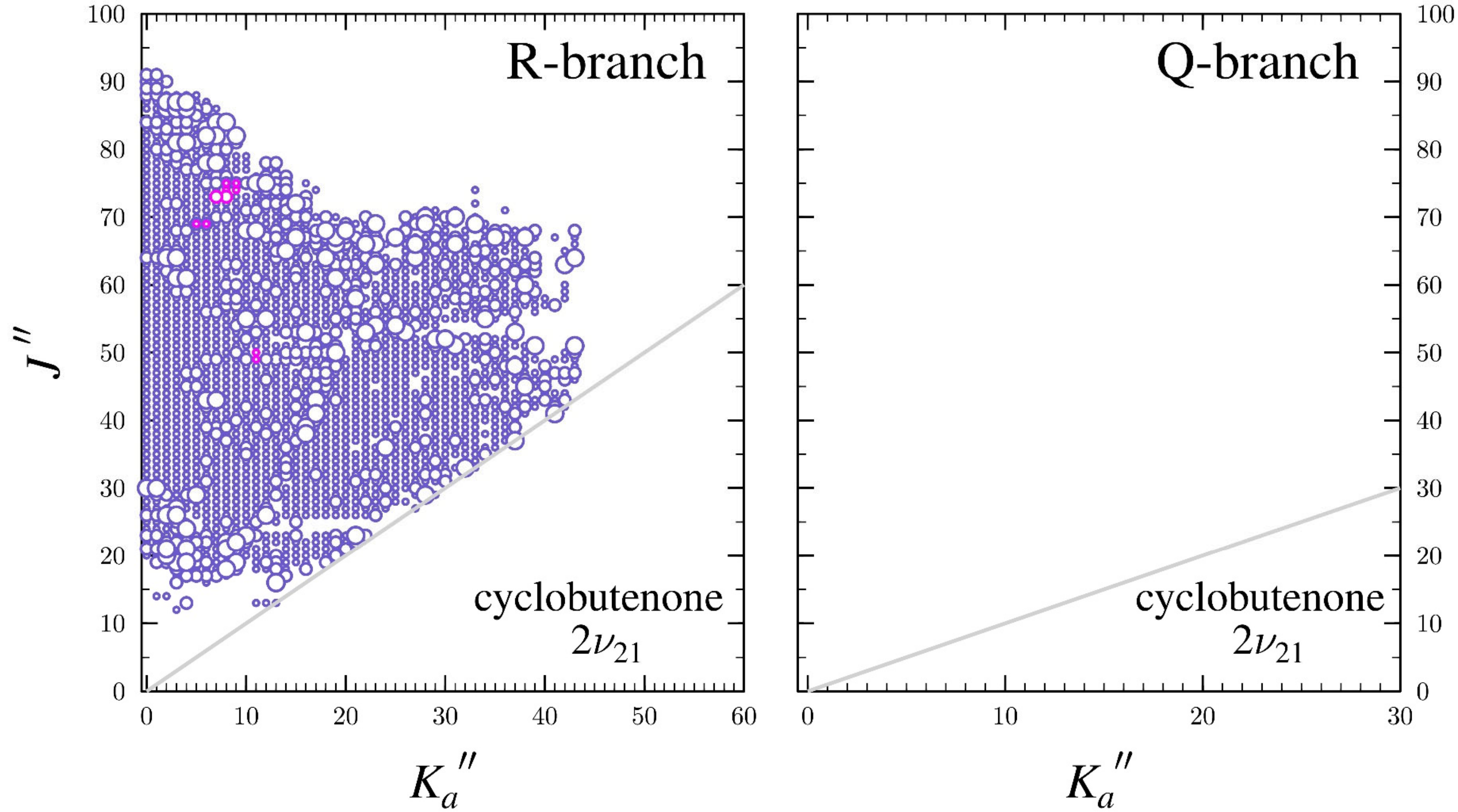

Figure S8. Data distribution plot for the least-squares fit of millimeter-wave spectroscopic data for cyclobutenone, $2\nu_{21}$. Slate blue circles are within-state transitions and magenta circles represent nominal interstate transitions with ν_{14}. All measurements are from the current work. The size of the outlined circle is proportional to the value of $|(f_{obs.} - f_{calc.})/\delta f|$, where δf is the frequency measurement uncertainty (50 kHz), and no quotient values are larger than three.

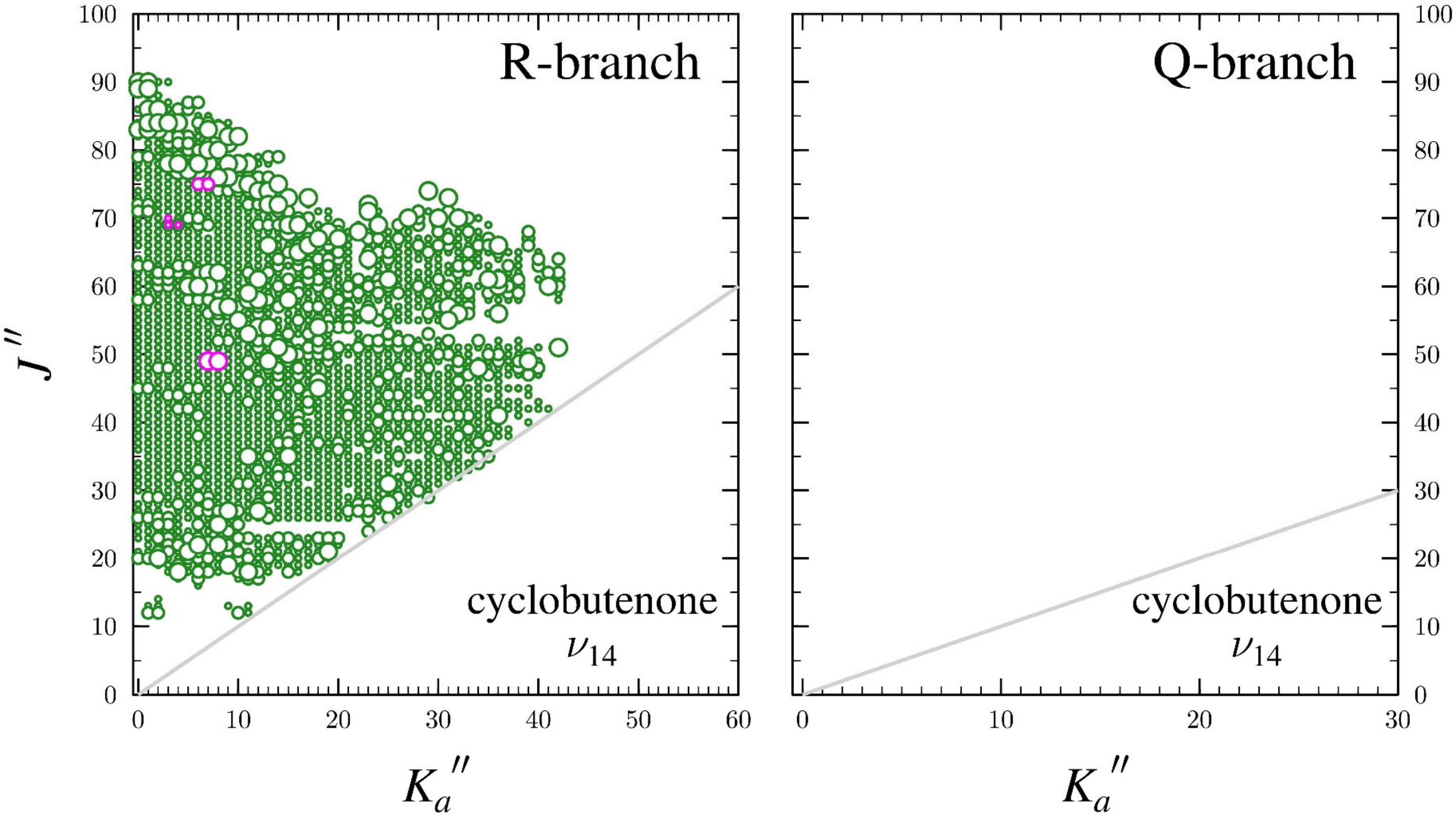

Figure S9. Data distribution plot for the least-squares fit of millimeter-wave spectroscopic data for cyclobutenone, ν_{14}. Green circles are within-state transitions and magenta circles represent nominal interstate transitions with $2\nu_{21}$. All measurements are from the current work. The size of the outlined circle is proportional to the value of $|(f_{obs.} - f_{calc.})/\delta f|$, where δf is the frequency measurement uncertainty (50 kHz), and no quotient values are larger than three.

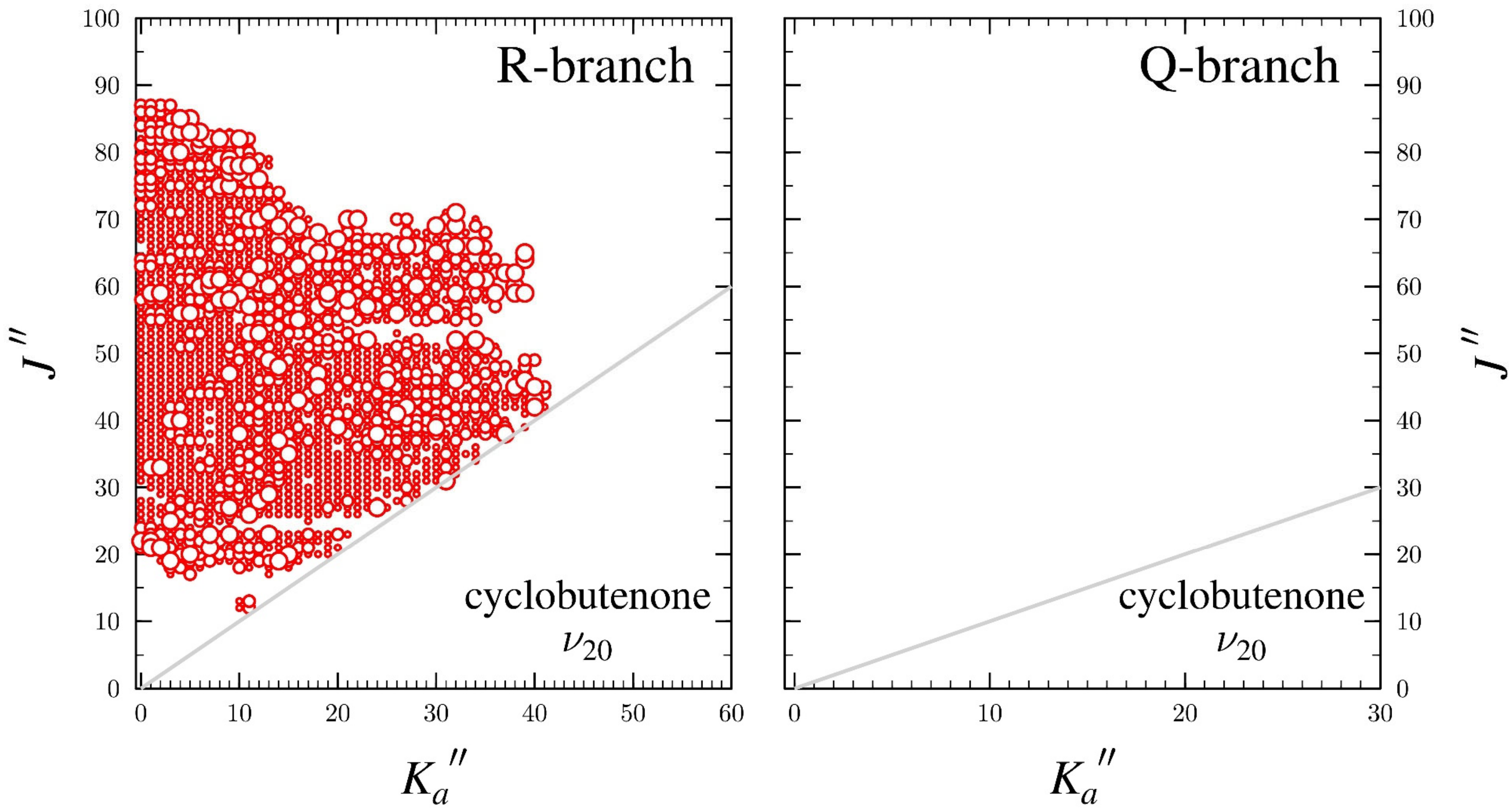

Figure S10. Data distribution plot for the least-squares fit of millimeter-wave spectroscopic data for cyclobutenone, ν_{20}. Red (all) circles are measurements from the current work. The size of the outlined circle is proportional to the value of $|(f_{obs.} - f_{calc.})/\delta f|$, where δf is the frequency measurement uncertainty (50 kHz), and no quotient values are larger than three.

Table S8. Determinable rotational constants calculated from A and S reductions, averaged determinable constants, and differences between determinable constants calculated from A and S reductions

	C_4H_4O	[1-^{13}C]-	[2-^{13}C]-	[3-^{13}C]-	[4-^{13}C]-	[1-^{18}O]-
$A_0''^{(A)}$ (MHz)	12548.270 05	12548.768 79	12219.062 08	12546.365 53	12187.434 69	12547.793 39
$B_0''^{(A)}$ (MHz)	5307.566 601	5290.953 363	5291.748 458	5189.194 048	5292.248 168	4993.407 706
$C_0''^{(A)}$ (MHz)	3820.184 16	3811.635 511	3780.996 083	3758.324 436	3778.194 672	3654.603 073
$A_0''^{(S)}$ (MHz)	12548.270 19	12548.771 47	12219.064 22	12546.367 76	12187.436 81	12547.805 75
$B_0''^{(S)}$ (MHz)	5307.566 659	5290.953 558	5291.748 724	5189.194 281	5292.248 316	4993.407 375
$C_0''^{(S)}$ (MHz)	3820.184 207	3811.635 445	3780.996 006	3758.324 385	3778.194 621	3654.602 935
Ave. A_0'' (MHz)	12548.270 12	12548.770 1	12219.063 1	12546.366 6	12187.435 7	12547.800
Ave. B_0'' (MHz)	5307.566 63	5290.953 46	5291.748 59	5189.194 16	5292.248 24	4993.407 54
Ave. C_0'' (MHz)	3820.184 18	3811.635 48	3780.996 04	3758.324 41	3778.194 65	3654.603 00
$A_0''^{(S)} - A_0''^{(A)}$ (MHz)	0.000 13	0.002 7	0.002 1	0.002 2	0.002 1	0.012
$B_0''^{(S)} - B_0''^{(A)}$ (MHz)	0.000 06	0.000 19	0.000 27	0.000 23	0.000 15	−0.000 33
$C_0''^{(S)} - C_0''^{(A)}$ (MHz)	0.000 05	−0.000 07	−0.000 08	−0.000 05	−0.000 05	−0.000 14

Table S9. Differences between experimental determinable constants and those predicted by *xrefit* [a]

Isotopologue	$A_0'' - A_{xrefit}''$ (MHz)	$B_0'' - B_{xrefit}''$ (MHz)	$C_0'' - C_{xrefit}''$ (MHz)
C_4H_4O	0.0442	0.0029	−0.0104
[1-^{13}C]	0.0187	0.0092	0.0044
[2-^{13}C]	0.0018	−0.0022	−0.0012
[3-^{13}C]	0.0095	0.0012	0.0012
[4-^{13}C]	−0.0003	−0.0027	−0.0015
[^{18}O]	−0.0274	−0.0005	0.0037

[a] B_0'' = the averaged (between A and S reductions) experimental determinable constant

Synthetic Details

This procedure is adapted from Ross *et al.*[14] Authentic spectra are also available from that work.

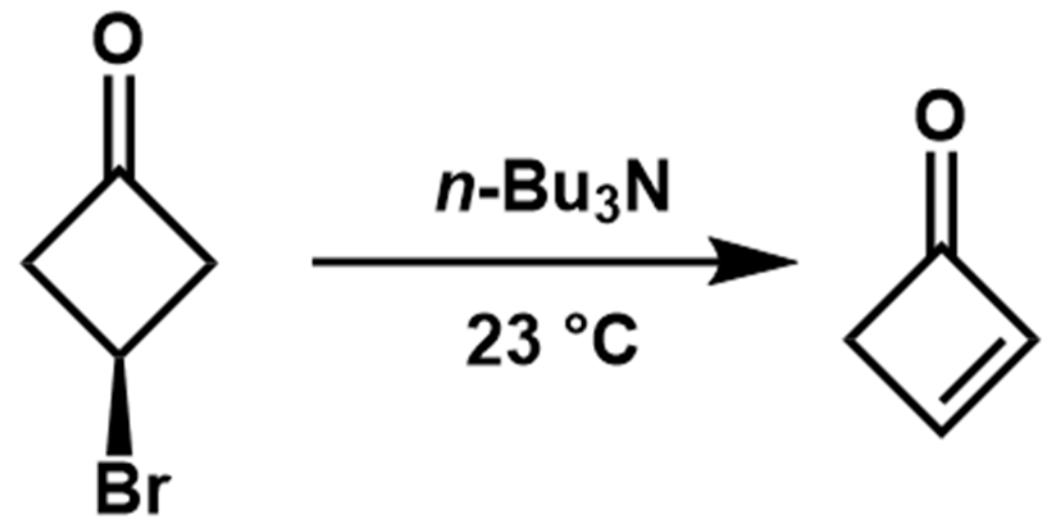

All glassware in this experiment was flame-dried and cooled under nitrogen gas prior to use. Tributylamine was purchased from Oakwood Chemical, 3-bromocyclobutanone was purchased from Ambeed, diethyl carbitol (*aka* diethylene glycol diethyl ether) was purchased from Sigma-Aldrich. All were used as received.

A 100-mL, single-neck, round-bottom flask equipped with a Teflon-coated magnetic stir bar is charged with tri-*n*-butylamine (32 mL, 135 mmol, 2 equiv, clear liquid). Neat 3-bromocyclobutanone (10.04 g, 67 mmol, 1 equiv, clear, yellow-tinted liquid) is added to the amine dropwise over 30 min and settles into a lower layer. The reaction mixture is stirred for 1 h at ambient temperature.

A 50-mL, single-neck, heart-shaped flask is connected to the reaction flask via a short path distillation head with Vigreux indents. The receiving flask is cooled to −78 °C in a dry ice/acetone bath, and the reaction flask is placed in an oil bath held constant at 25 °C. As the reaction flask is vigorously stirred, pressure is carefully reduced to 2.5 mmHg and held for 20 min to ensure that cyclobutenone does not foam over too rapidly. At this time the receiving flask contains semi-frozen cyclobutenone, which rapidly melts to a clear liquid upon moving the receiving flask to ambient temperature (1.76 g, 39% yield, over two fractions). In this preparation, both fractions of crude melted cyclobutenone are dissolved in diethyl carbitol (50 mL total) for storage as a 0.5 M solution. The final solution is cloudy white, with some suspended white solid, indicating partial polymerization prior to dilution. Prior to addition of diethyl carbitol, product purity was checked *via* ^{1}H-NMR. The product was approx. 98% pure, the other 2% being tributylamine which has a significantly lower vapor pressure than does cyclobutenone and does not interfere with rotational spectroscopy, assuming careful vacuum control.

The product solution can be stored in a freezer (−20 ℃) for at least one month. At room temperature, the product may demonstrate yellowing and/or increased precipitate formation, but has still been viable for rotational spectroscopic analysis even after significant yellowing.

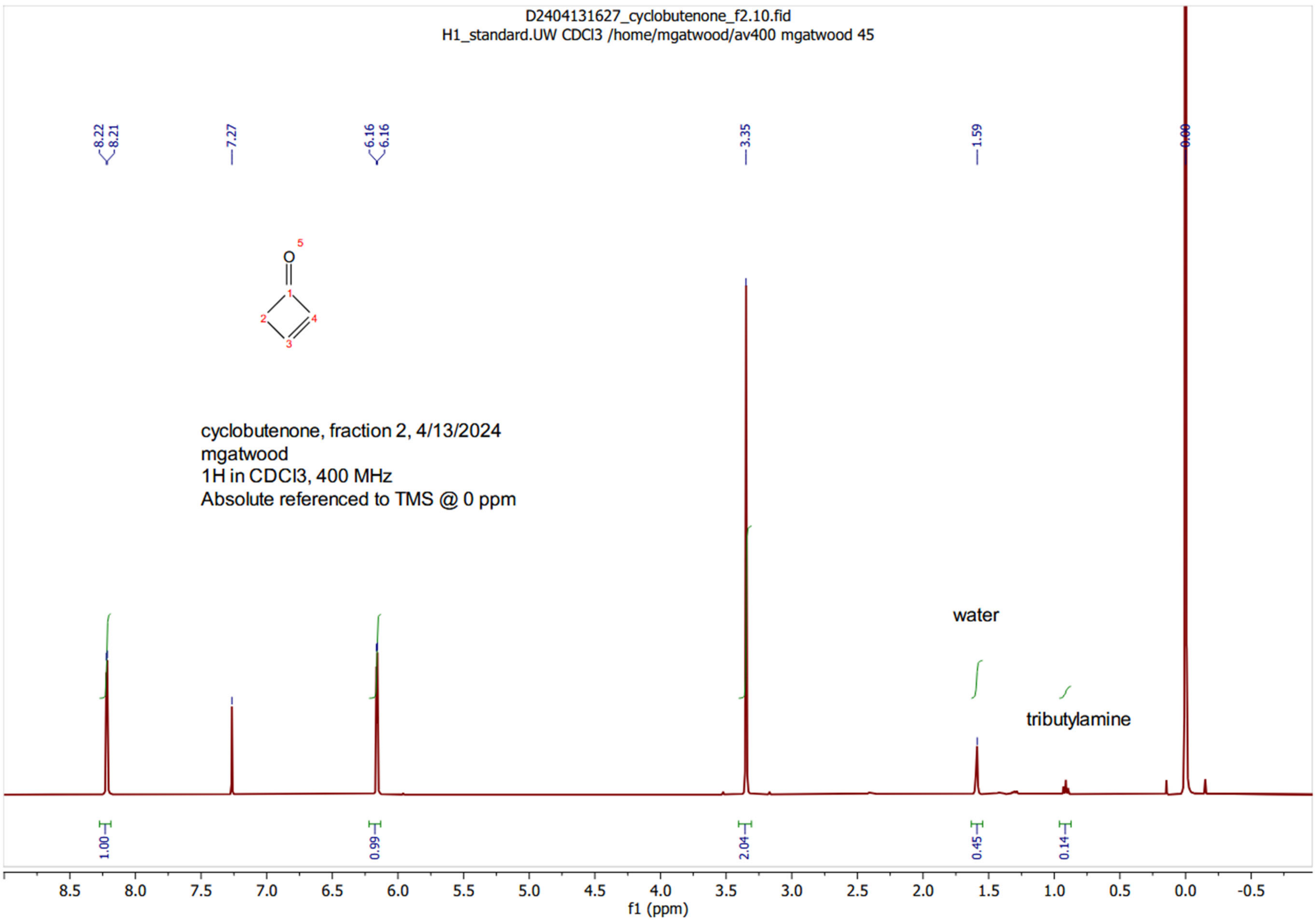
D2404131627_cyclobutenone_f2.10.fid
H1_standard.UW CDCl3 /home/mgatwood/av400 mgatwood 45
8.22
8.21
7.27
6.16
6.16
3.35
1.59
0.00
cyclobutenone, fraction 2, 4/13/2024
mgatwood
1H in CDCl3, 400 MHz
Absolute referenced to TMS @ 0 ppm
water
tributylamine
1.00
0.99
2.04
0.45
0.14
8.5
8.0
7.5
7.0
6.5
6.0
5.5
5.0
4.5
4.0
3.5
3.0
2.5
2.0
1.5
1.0
0.5
0.0
-0.5
f1 (ppm)

References

(1) Gordy, W.; Cook, R., *Microwave Molecular Spectra*. 3rd. ed.; Wiley Interscience: New York, 1984.

(2) Morgan, W. J.; Matthews, D. A.; Ringholm, M.; Agarwal, J.; Gong, J. Z.; Ruud, K.; Allen, W. D.; Stanton, J. F.; Schaefer, H. F., III, Geometric Energy Derivatives at the Complete Basis Set Limit: Application to the Equilibrium Structure and Molecular Force Field of Formaldehyde *J. Chem. Theory Comput.* **2018**, *14*, 1333−1350.

(3) Puzzarini, C.; Bloino, J.; Tasinato, N.; Barone, V., Accuracy and Interpretability: The Devil and the Holy Grail. New Routes across Old Boundaries in Computational Spectroscopy. *Chem. Rev.* **2019**, *119*, 8131-8191.

(4) Feller, D., The use of systematic sequences of wave functions for estimating the complete basis set, full configuration interaction limit in water *J. Chem. Phys.* **1993**, *98*, 7059-7071.

(5) Bomble, Y. J.; Stanton, J. F.; Kallay, M.; Gauss, J., Coupled-Cluster Methods Including Noniterative Corrections for Quadruple Excitations. *J. Chem. Phys.* **2005**, *123*, 054101.

(6) Cheng, L.; Gauss, J., Analytic energy gradients for the spin-free exact two-component theory using an exact block diagonalization for the one-electron Dirac Hamiltonian. *J. Chem. Phys.* **2011**, *135*, 084114.

(7) Dyall, K. G., Interfacing relativistic and nonrelativistic methods. II. Investigation of a low-order approximation. *J. Chem. Phys.* **1998**, *109*, 4201-4208.

(8) Liu, W.; Peng, D., Exact two-component Hamiltonians revisited. *J. Chem. Phys.* **2009**, *131*, 031104.

(9) Born, M.; Huang, K., *Dynamical Theory of Crystal Lattices*. Oxford Univ. Press: 1954; p 430.

(10) Handy, N. C.; Yamaguchi, Y.; Schaefer, H. F., The diagonal correction to the Born–Oppenheimer approximation: Its effect on the singlet–triplet splitting of CH_2 and other molecular effects. *J. Chem. Phys.* **1986**, *84*, 4481-4484.

(11) Gauss, J.; Puzzarini, C., Quantum-chemical calculation of Born-Oppenheimer breakdown parameters to rotational constants. *Mol. Phys.* **2010**, *108*, 269-277.

(12) Puzzarini, C.; Gauss, J., Quantum-chemical determination of Born-Oppenheimer breakdown parameters for rotational constants: the open-shell species CN, CO^+ and BO *Mol. Phys.* **2013**, *111*, 2204-2210.

(13) Carrillo, M. J.; Marasinghe, D.; Feeley, E. B.; Sobie, K. M.; Zarzycki, R. J.; Carter-Fenk, K.; Fenk, C. J.; Tubergen, M. J., Theoretical and Microwave Spectroscopic Characterization of Cyclobutenone: Planar or Puckered? *J. Phys. Chem A* **2023**, *127*, 9082-9087.

(14) Ross, A. G.; Li, X.; Danishefsky, S. J., Preparation of cyclobutenone. *Org. Synth.* **2012**, *89*, 491-500.
